# Supplementary material for: Protective mitochondrial fission induced by stress-responsive protein GJA1-20k
Source: eLife. 2021 Oct 5;10:e69207. doi: 10.7554/eLife.69207 (PMC8492060; doi:10.7554/eLife.69207)
Supplement: Source data 1. [file elife-69207-supp1.zip › Uncropped membrane/Uncropped membrane_Revision.pdf]

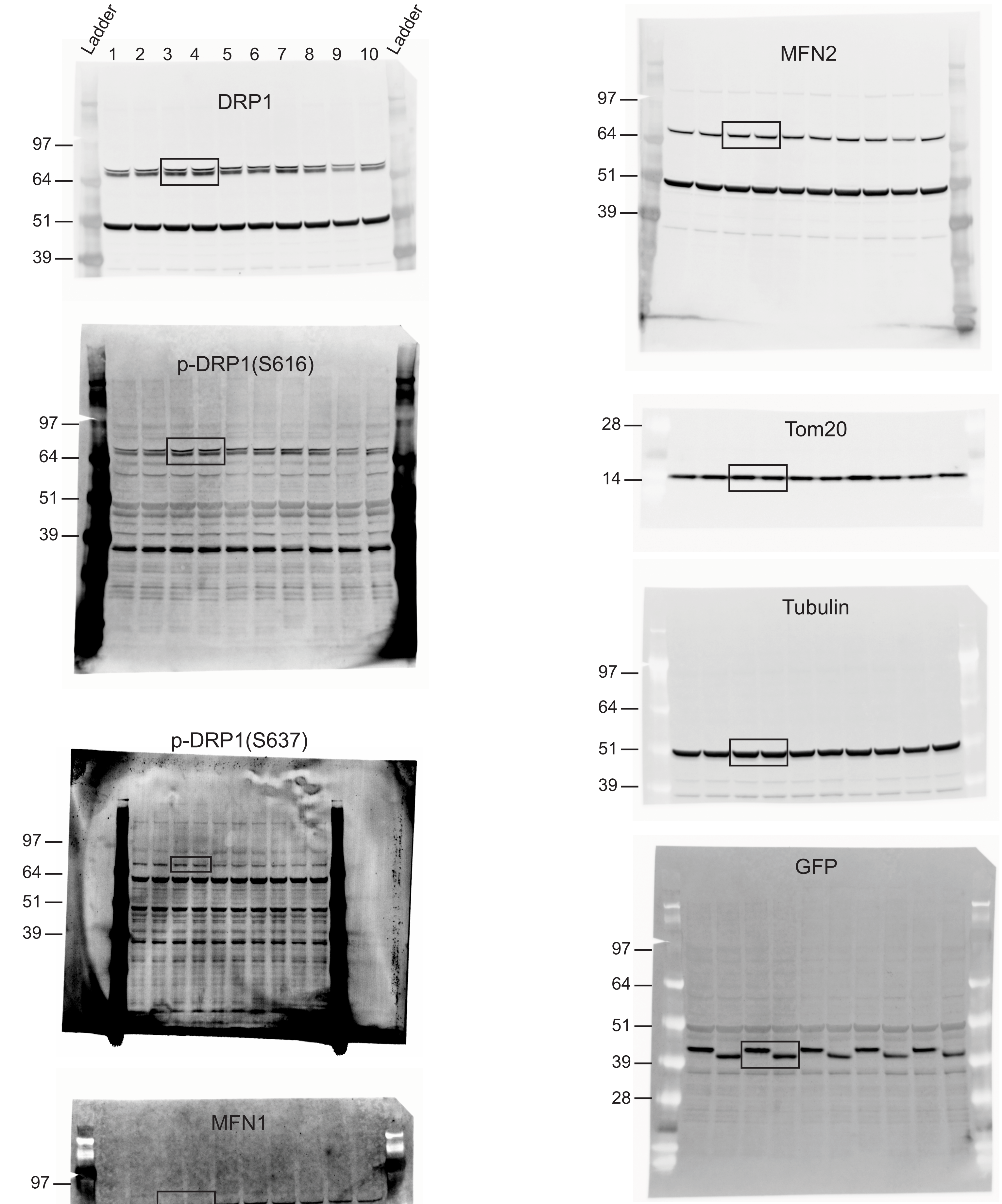

Lane
1: GST Sample #1
2: GJA1-20k Sample #1
3: GST Sample #2
4: GJA1-20k Sample #2
5: GST Sample #3
6: GJA1-20k Sample #3
7: GST Sample #4
8: GJA1-20k Sample #4
9: GST Sample #5
10: GJA1-20k Sample #5

Same order in all membrane

Uncropped membarane images. Surrounded area are used for Main Figure 2.

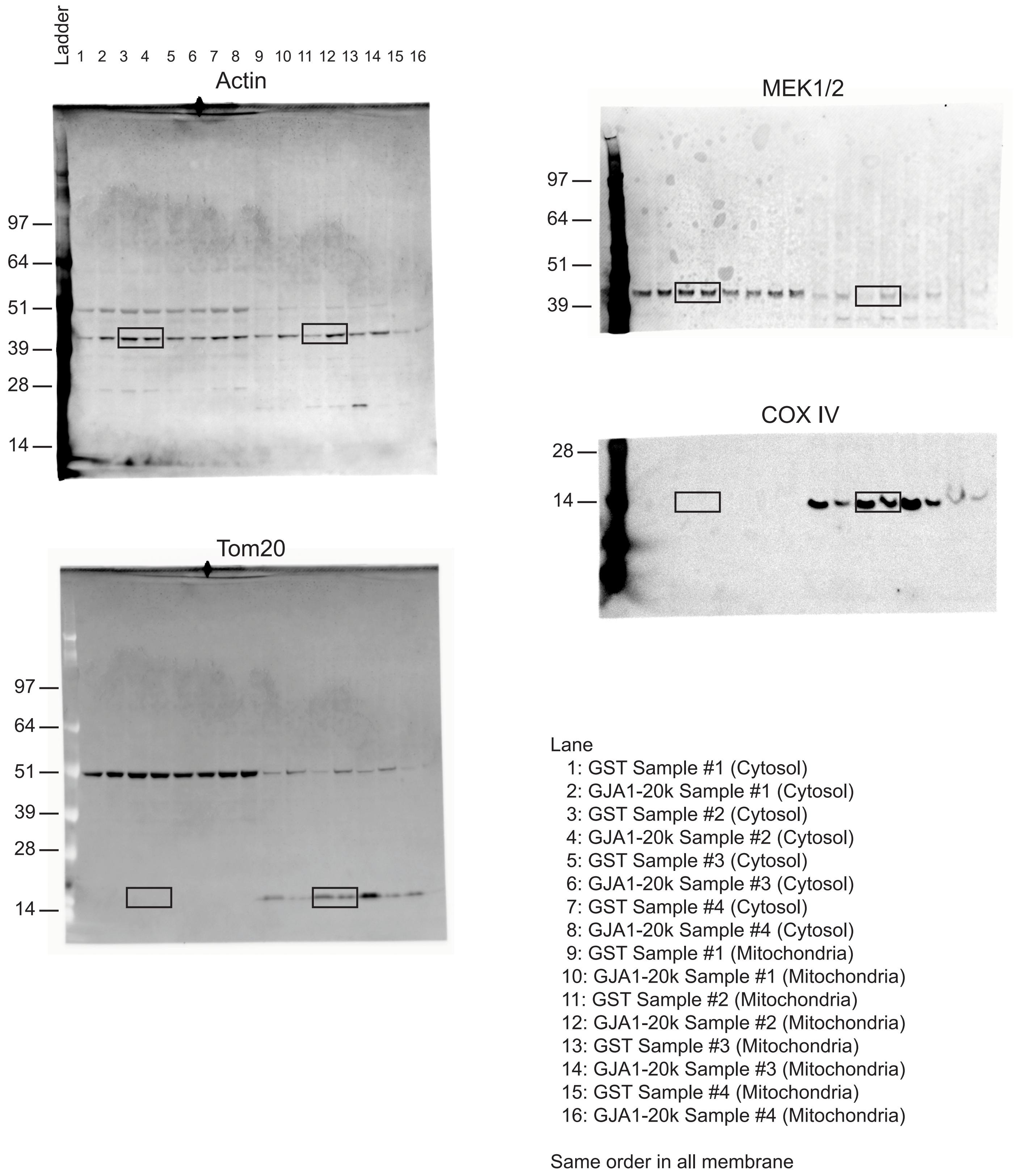

Uncropped membarane images. Surrounded area are used for Main Figure 3.

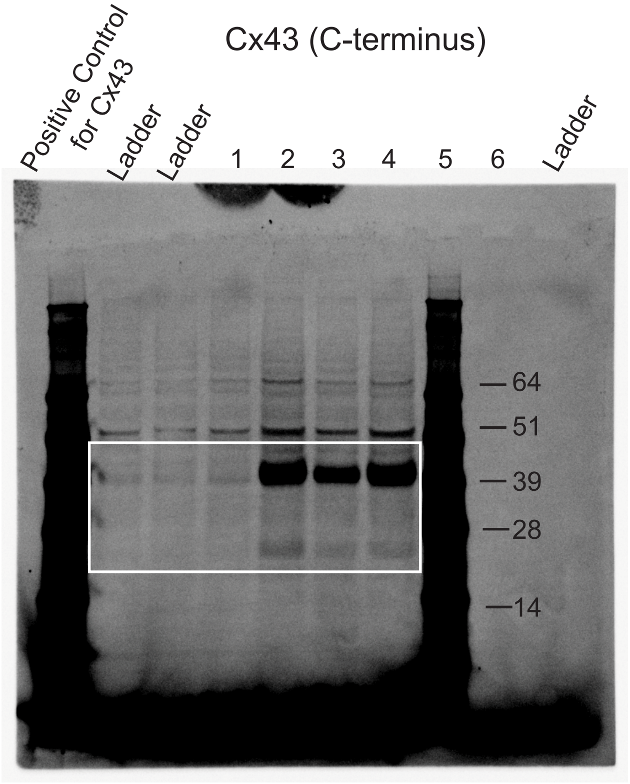

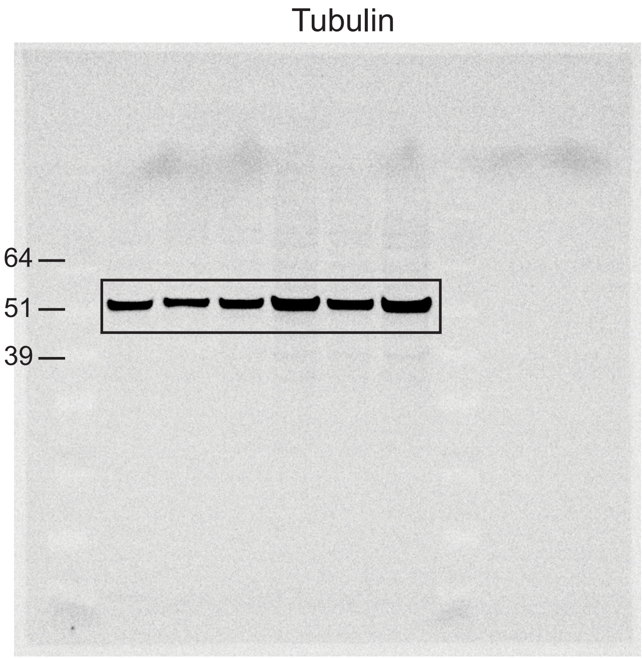

Lane
1: Gja1 siRNA Sample #1
2: Gja1 siRNA Sample #2
3: Gja1 siRNA Sample #3
4: Control siRNA Sample #1
5: Control siRNA Sample #2
6: Control siRNA Sample #3

Same order in all membrane

Uncropped membarane images. Surrounded area are used for Figure 1—figure supplement 1.

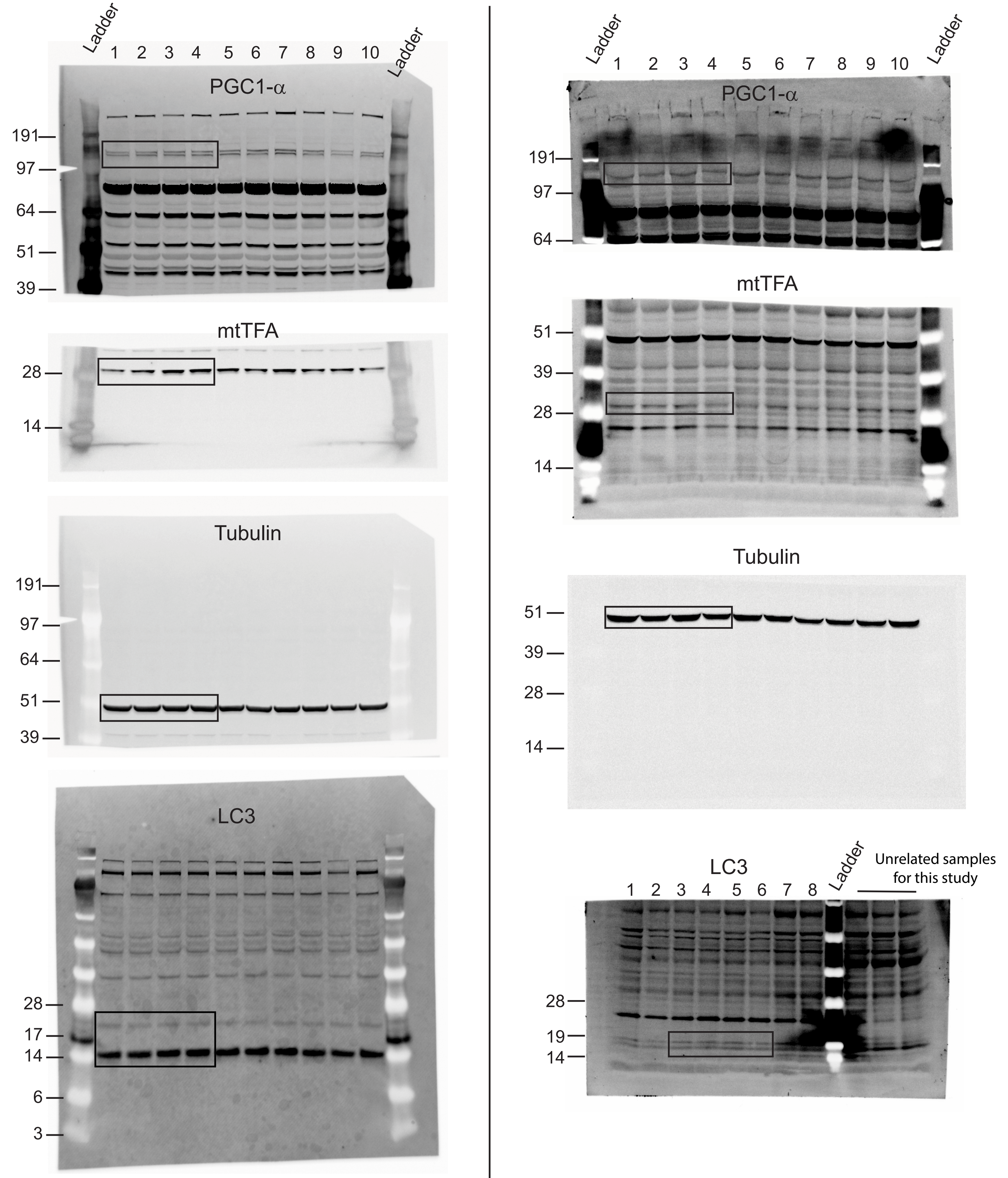

Lane

1: GST Sample #1
2: GJA1-20k Sample #1
3: GST Sample #2
4: GJA1-20k Sample #2
5: GST Sample #3
6: GJA1-20k Sample #3
7: GST Sample #4
8: GJA1-20k Sample #4
9: GST Sample #5
10: GJA1-20k Sample #5

Lane

1: WT Sample #1
2: Gja1$^{M213L/M231L}$ Sample #1
3: WT Sample #2
4: Gja1$^{M213L/M231L}$ Sample #2
5: WT Sample #3
6: Gja1$^{M213L/M231L}$ Sample #3
7: WT Sample #4
8: Gja1$^{M213L/M231L}$ Sample #4
9: WT Sample #5
10: Gja1$^{M213L/M231L}$ Sample #5

Uncropped membarane images. Surrounded area are used for Figure1—figure supplement 2.

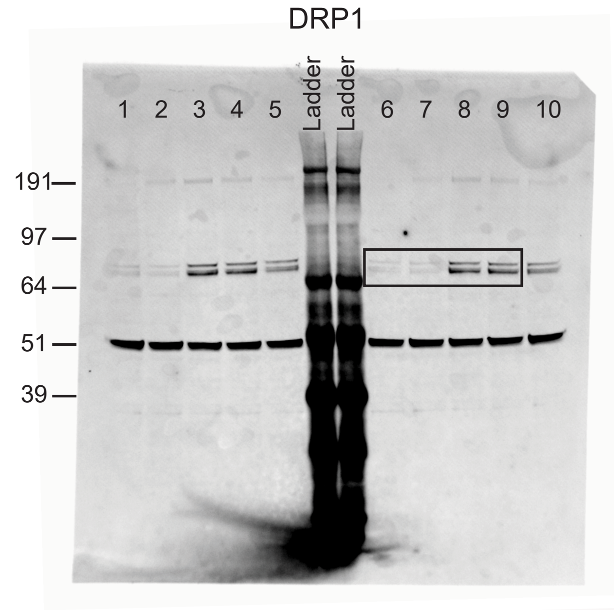

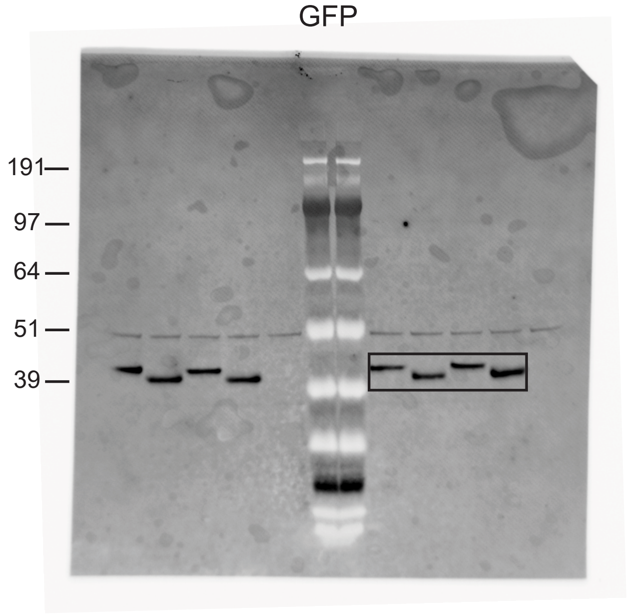

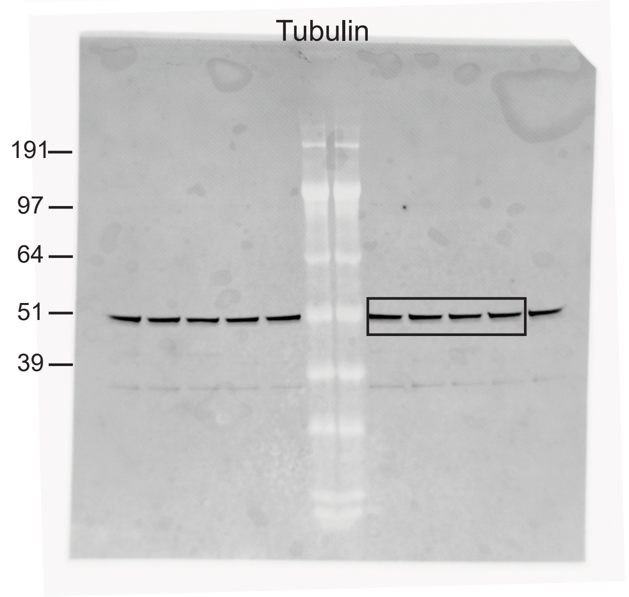

Lane

1: GST + DRP1 siRNA Sample #1
2: GJA1-20k + DRP1 siRNA Sample #1
3: GST + Control siRNA Sample #1
4: GJA1-20k + Control siRNA Sample #1
5: Blank Control (no transfection, no siRNA)
6: GST + DRP1 siRNA Sample #2
7: GJA1-20k + DRP1 siRNA Sample #2
8: GST + Control siRNA Sample #2
9: GJA1-20k + Control siRNA Sample #2
10: Blank Control (no transfection, no siRNA)

Same order in all membrane

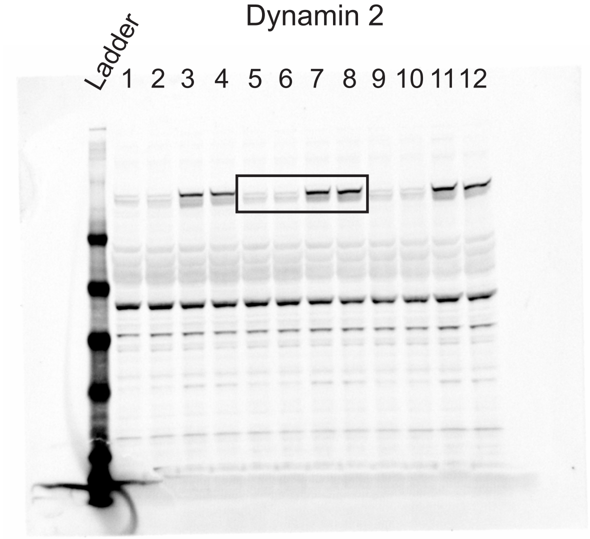

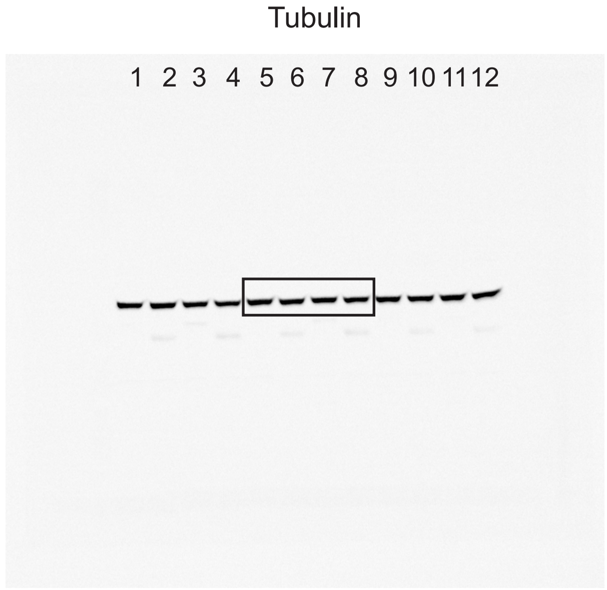

Lane

1: GST + DNM2 siRNA Sample #1
2: GJA1-20k + DNM2 siRNA Sample #1
3: GST + Control siRNA Sample #1
4: GJA1-20k + Control siRNA Sample #1
5: GST + DNM2 siRNA Sample #2
6: GJA1-20k + DNM2 siRNA Sample #2
7: GST + Control siRNA Sample #2
8: GJA1-20k + Control siRNA Sample #2
9: GST + DNM2 siRNA Sample #3
10: GJA1-20k + DNM2 siRNA Sample #3
11: GST + Control siRNA Sample #3
12: GJA1-20k + Control siRNA Sample #3

Uncropped membarane images. Surrounded area are used for Figure 2—figure supplement 1.
