# Supplementary figures and images for: Protective mitochondrial fission induced by stress-responsive protein GJA1-20k
Source: eLife. 2021 Oct 5;10:e69207. doi: 10.7554/eLife.69207 (PMC8492060; doi:10.7554/eLife.69207)

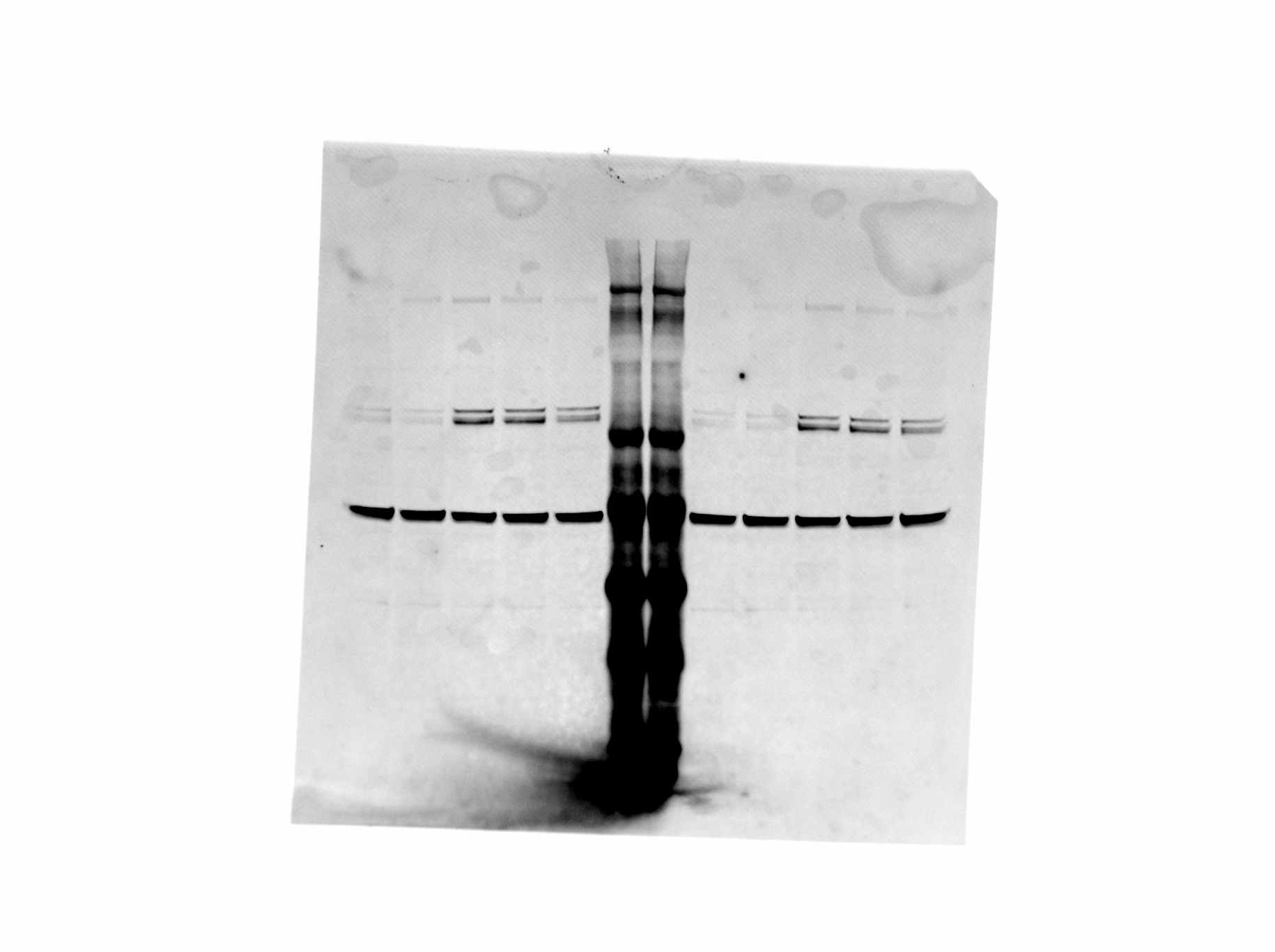

Supplement: Source data 1. [file elife-69207-supp1.zip › Uncropped membrane/Fig2-supplement/DRP1.tif]

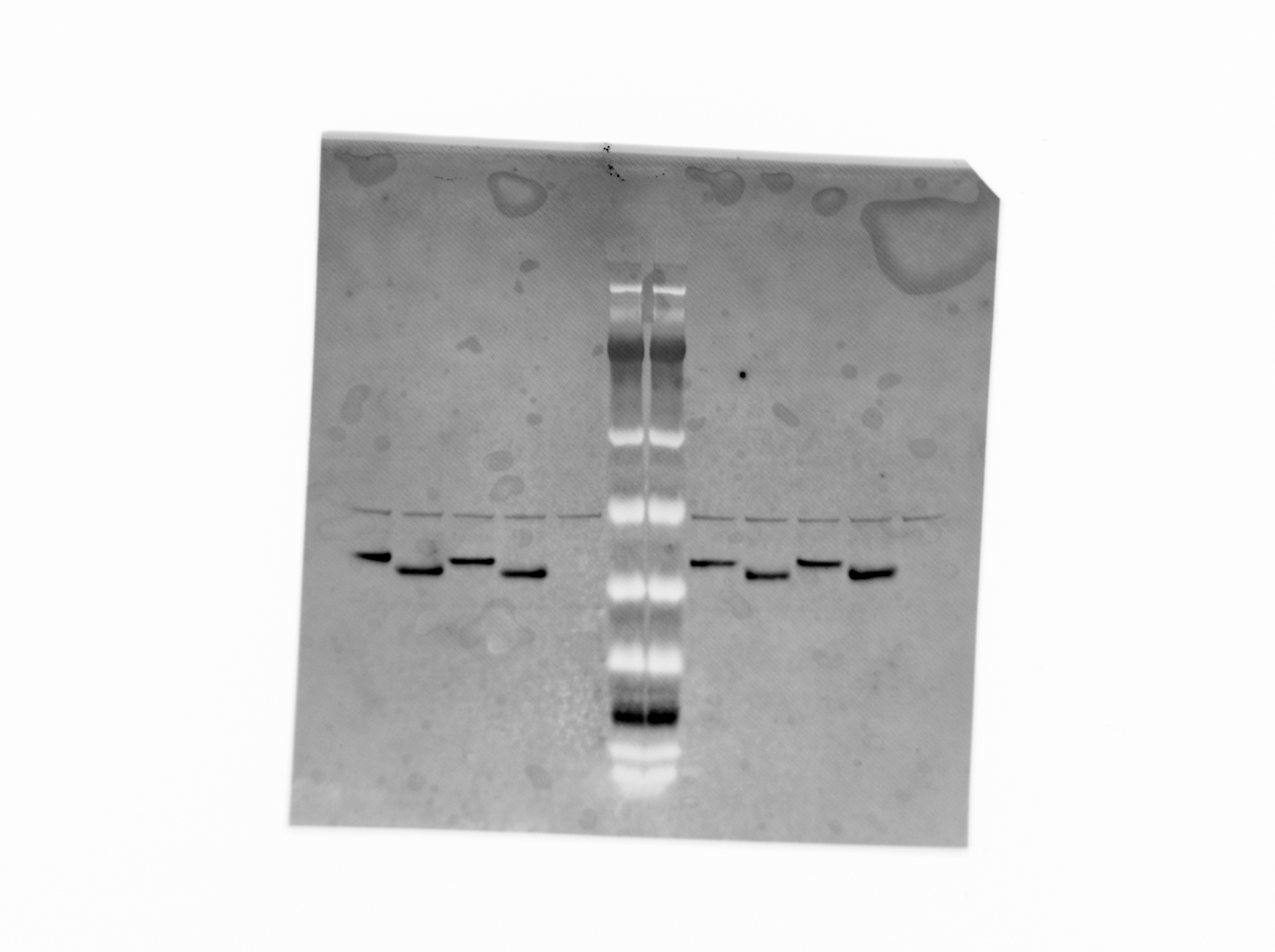

Supplement: Source data 1. [file elife-69207-supp1.zip › Uncropped membrane/Fig2-supplement/GFP.tif]

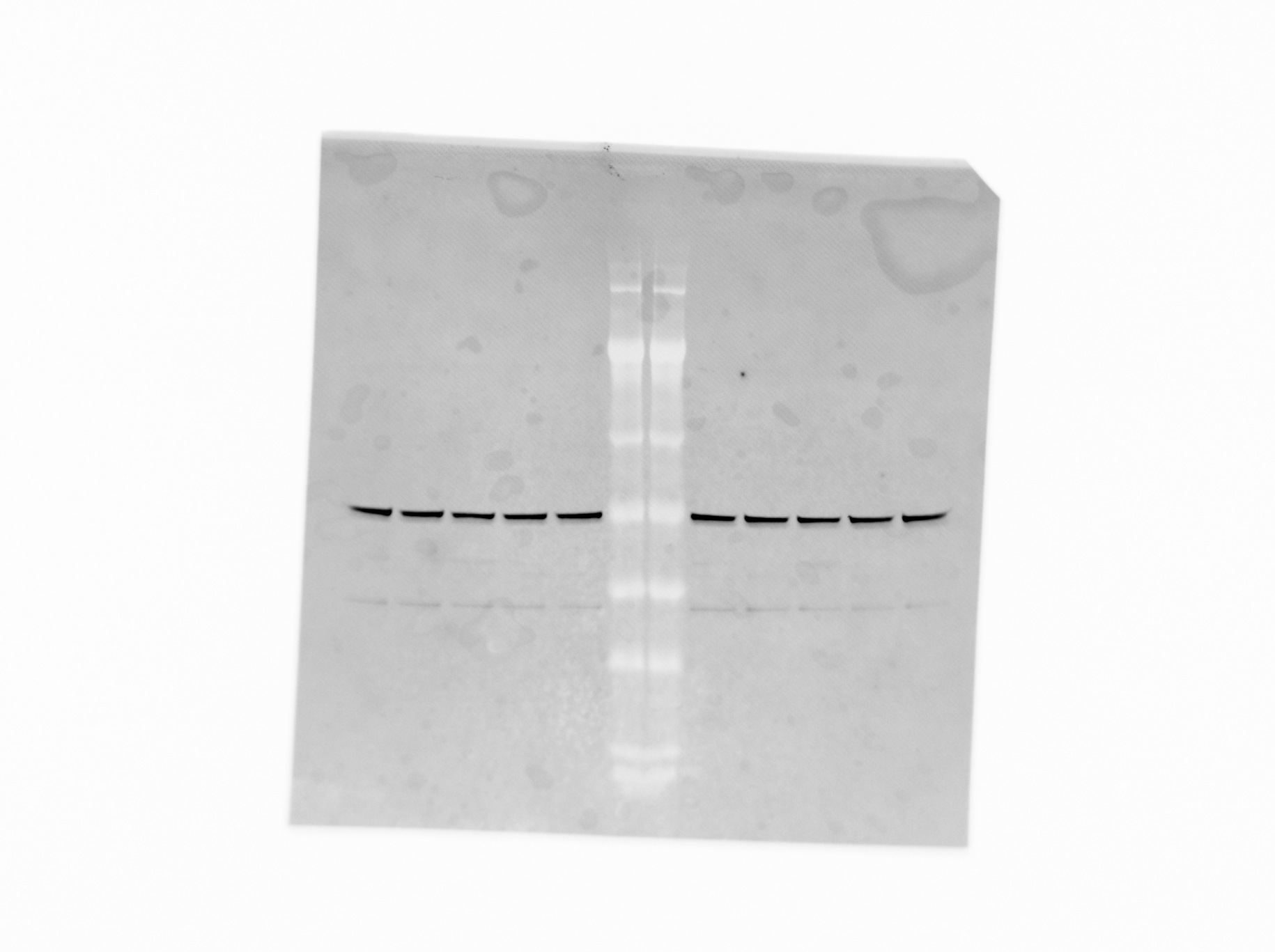

Supplement: Source data 1. [file elife-69207-supp1.zip › Uncropped membrane/Fig2-supplement/Tubulin.tif]

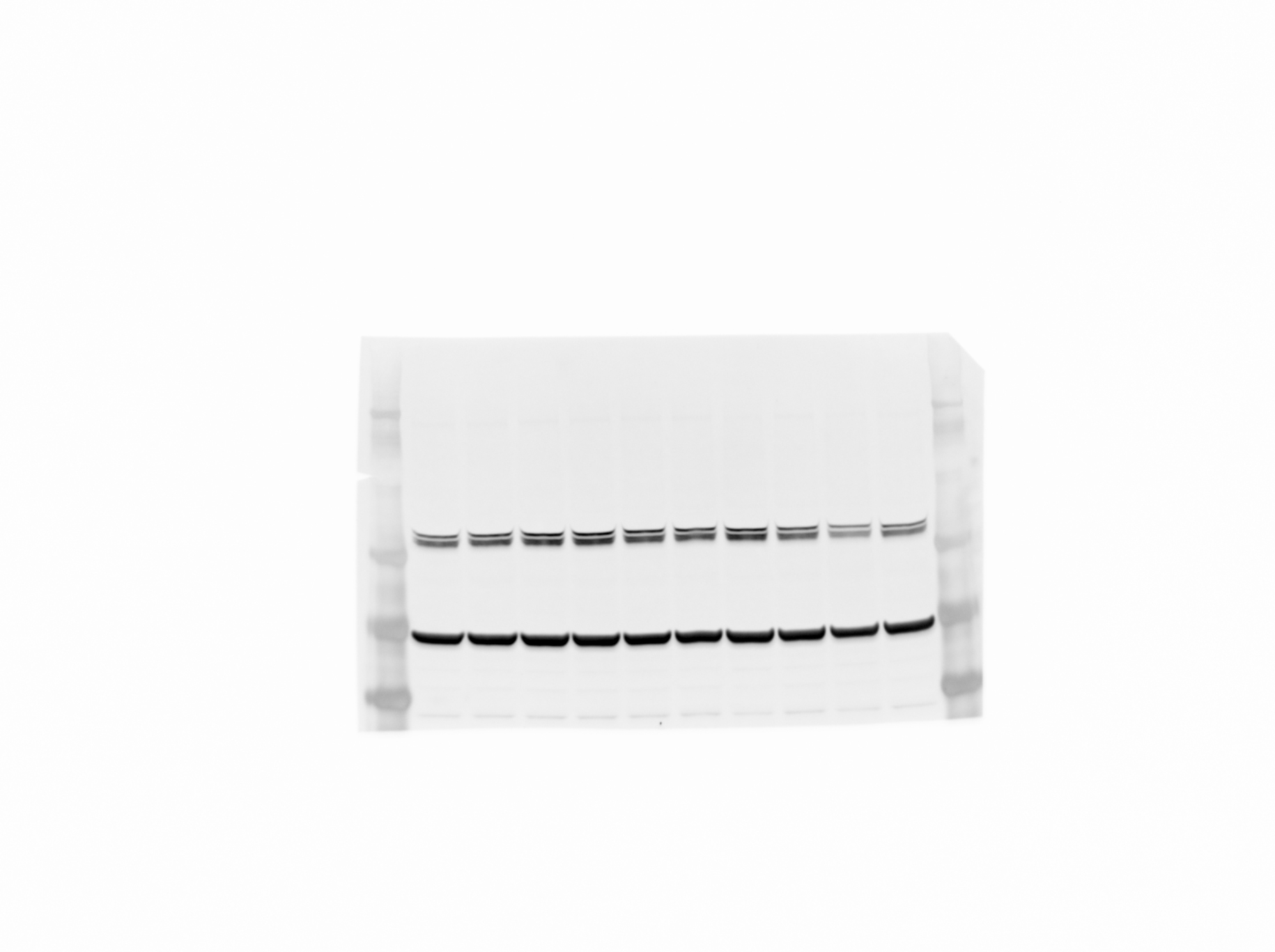

Supplement: Source data 1. [file elife-69207-supp1.zip › Uncropped membrane/Figure 2/DRP1.tif]

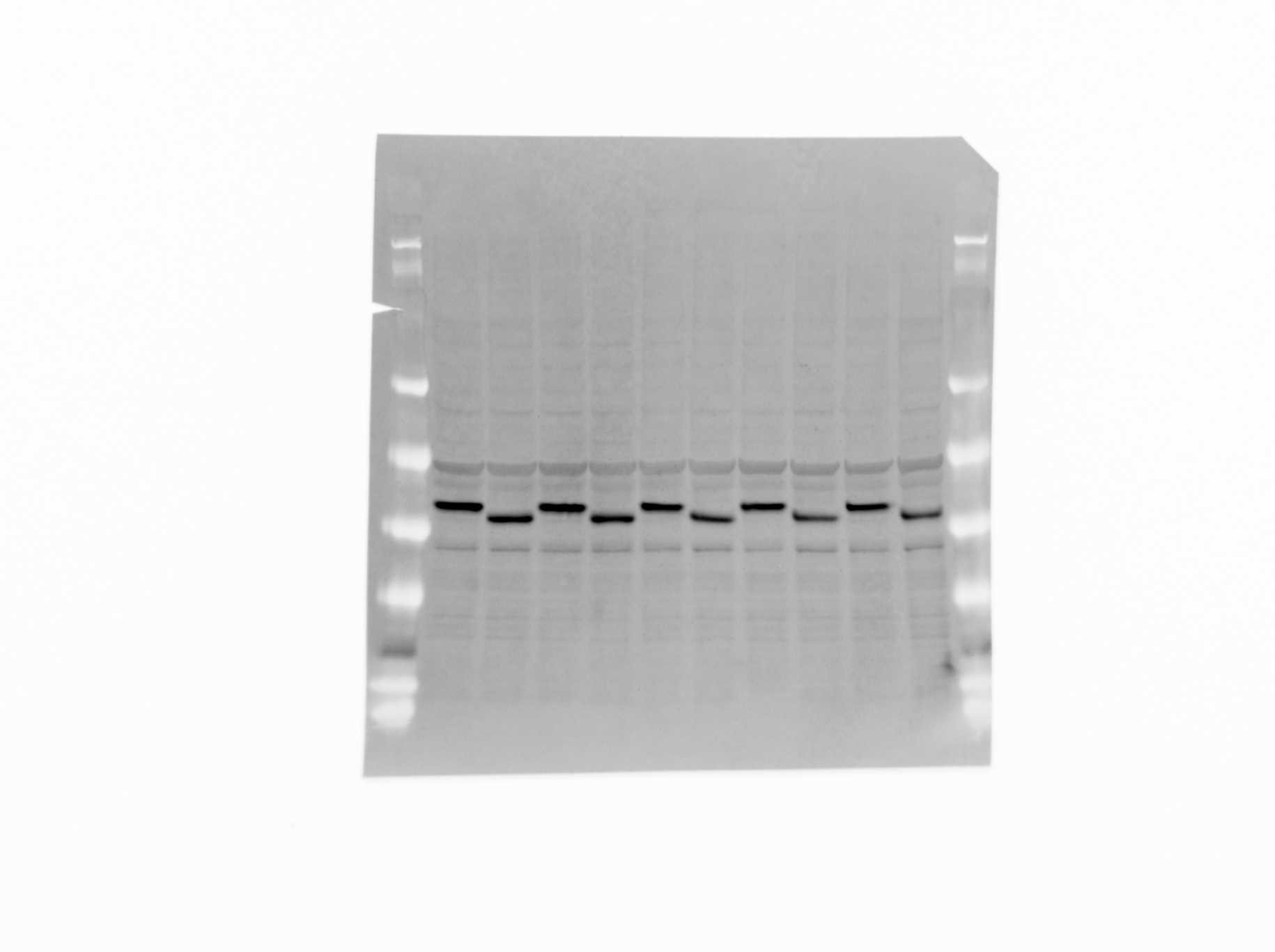

Supplement: Source data 1. [file elife-69207-supp1.zip › Uncropped membrane/Figure 2/GFP.tif]

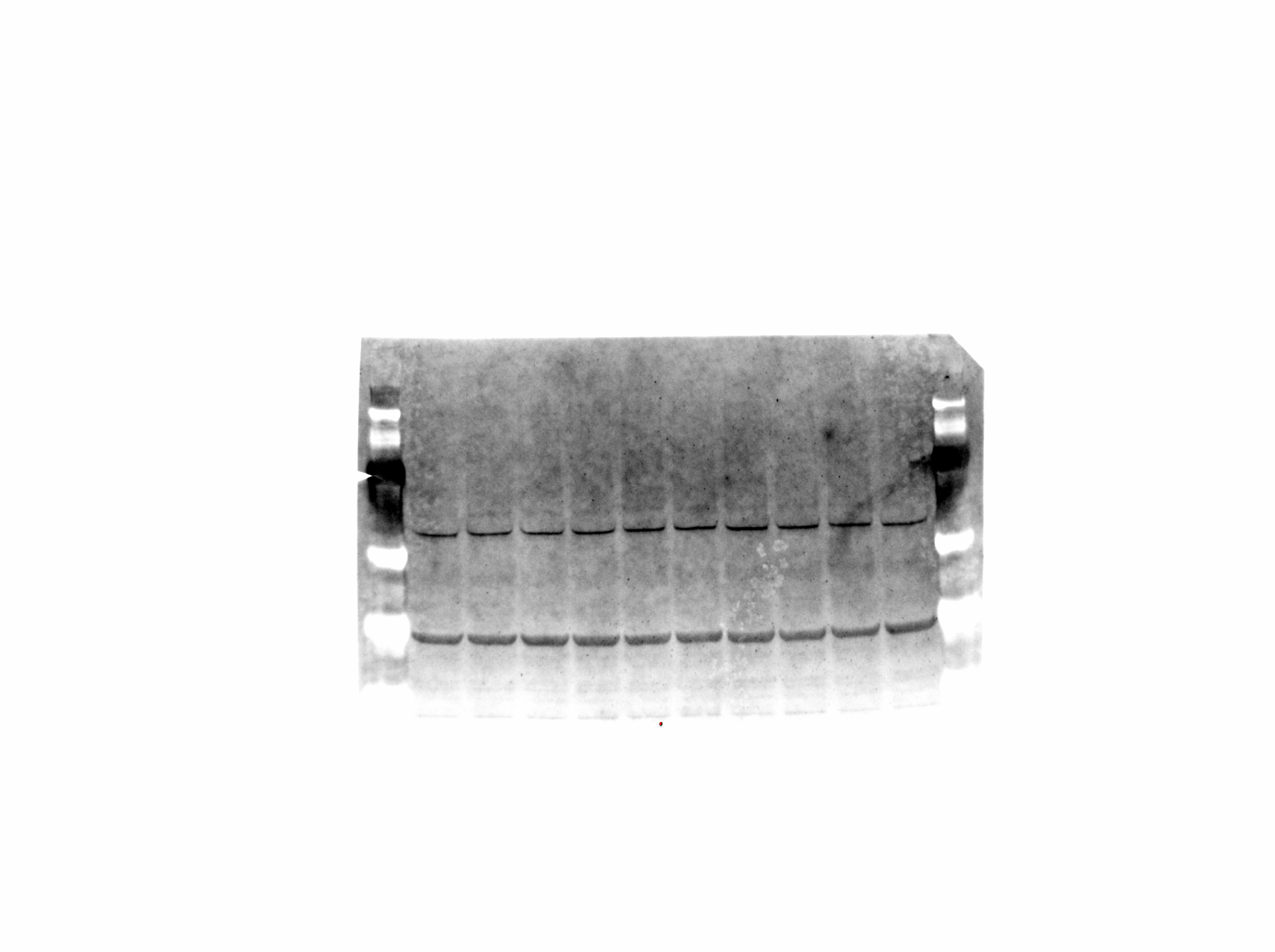

Supplement: Source data 1. [file elife-69207-supp1.zip › Uncropped membrane/Figure 2/MFN1.tif]

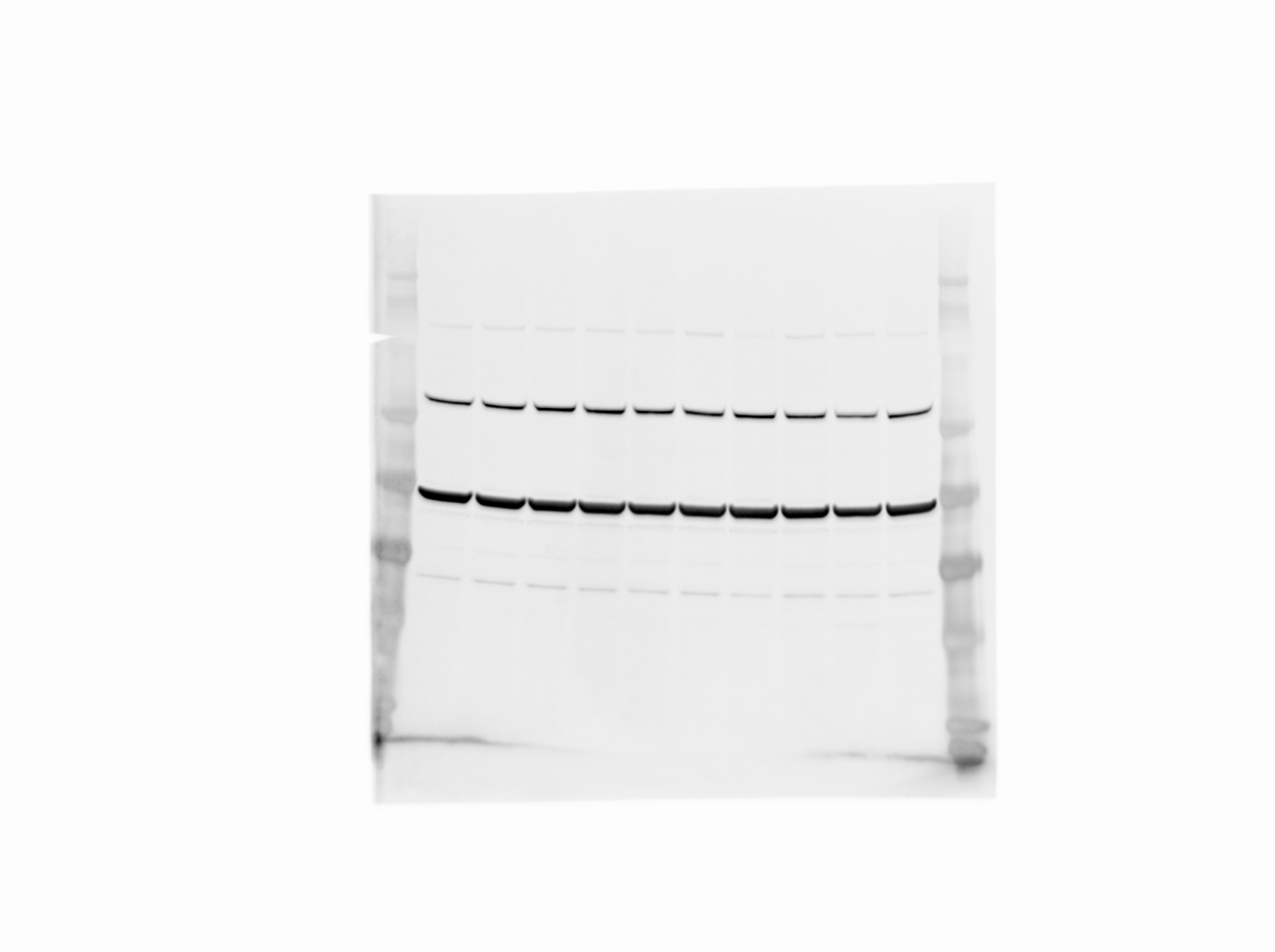

Supplement: Source data 1. [file elife-69207-supp1.zip › Uncropped membrane/Figure 2/MFN2.tif]

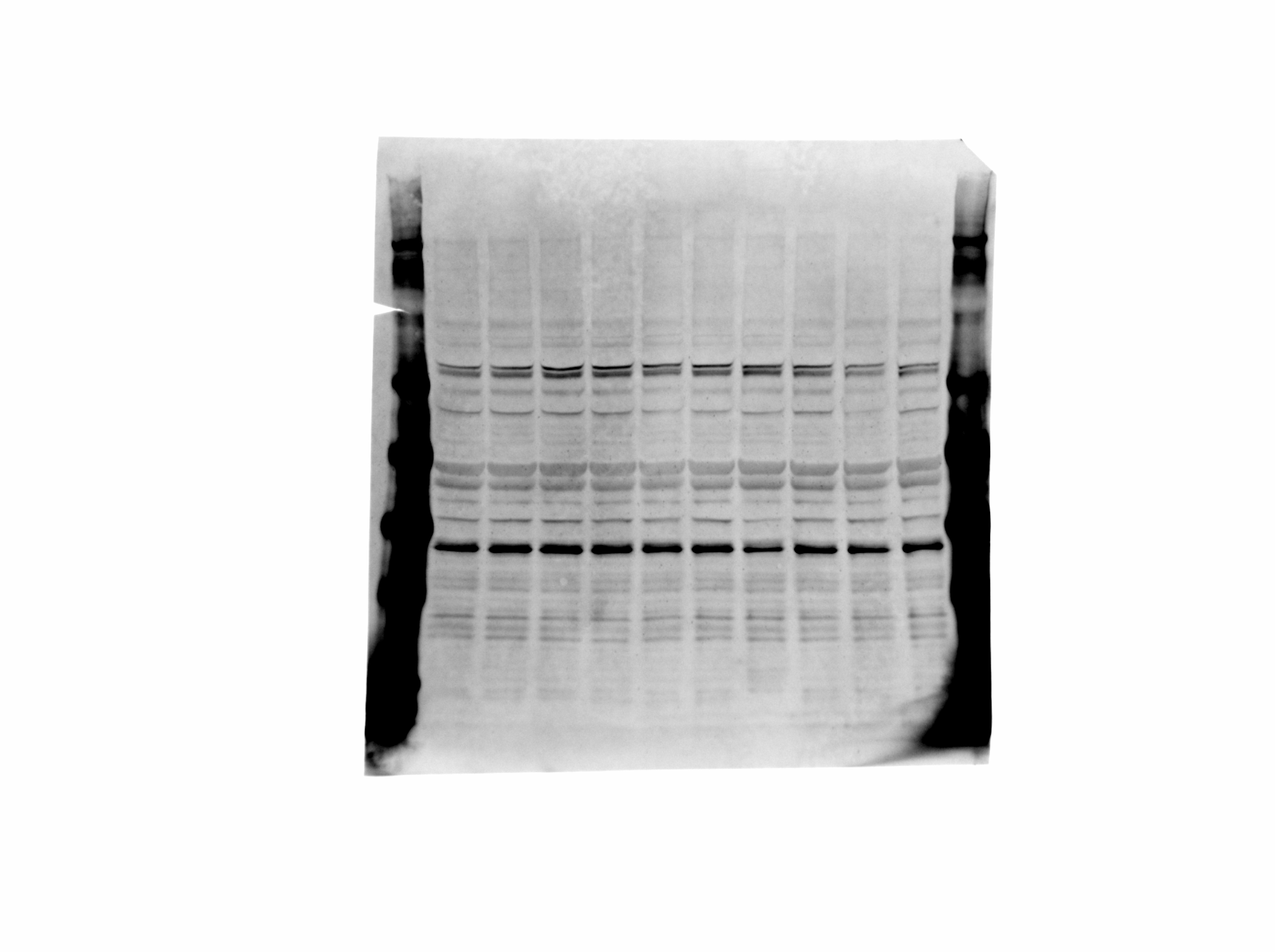

Supplement: Source data 1. [file elife-69207-supp1.zip › Uncropped membrane/Figure 2/p-DRP1(S616).tif]

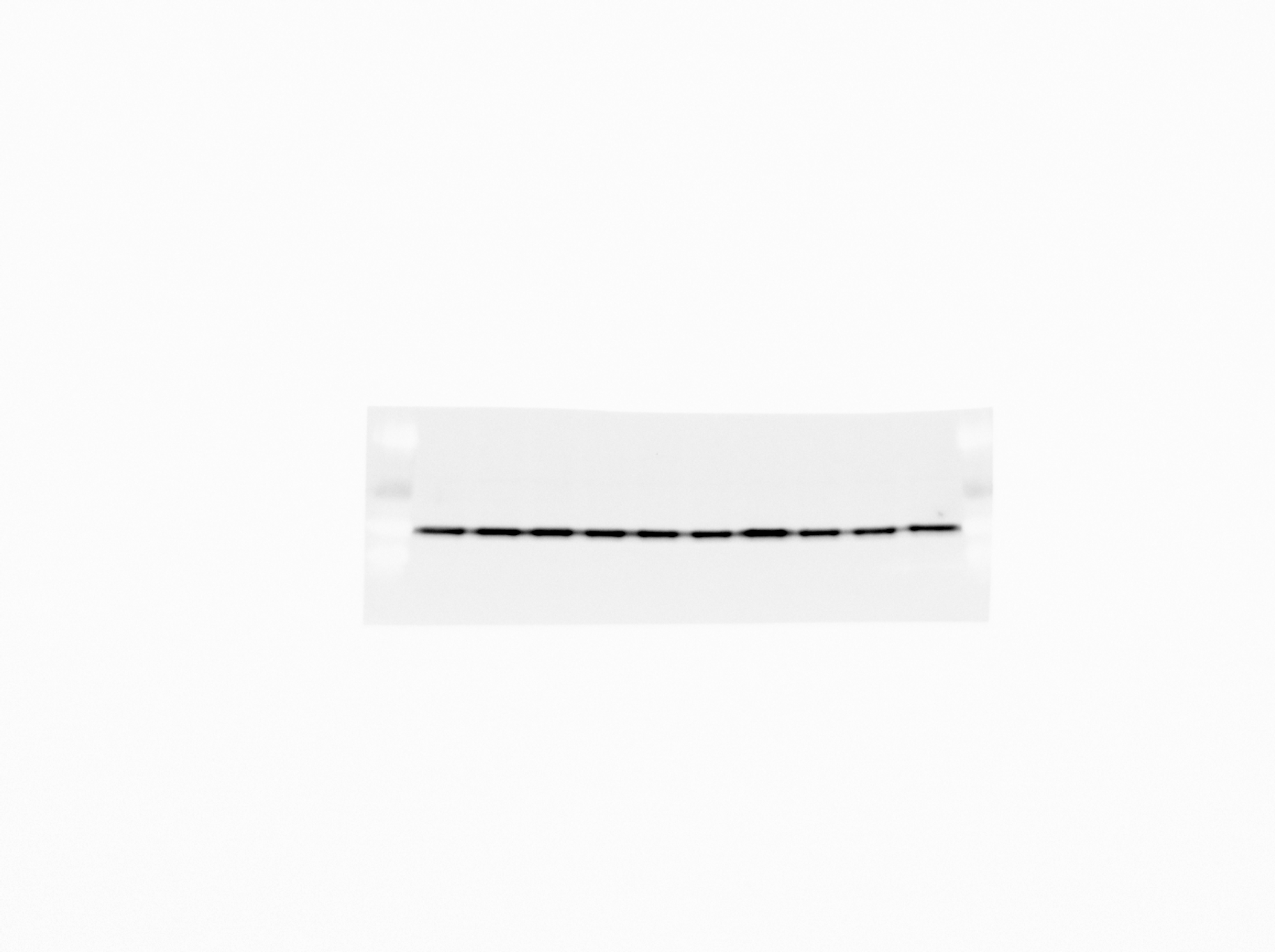

Supplement: Source data 1. [file elife-69207-supp1.zip › Uncropped membrane/Figure 2/Tom20.tif]

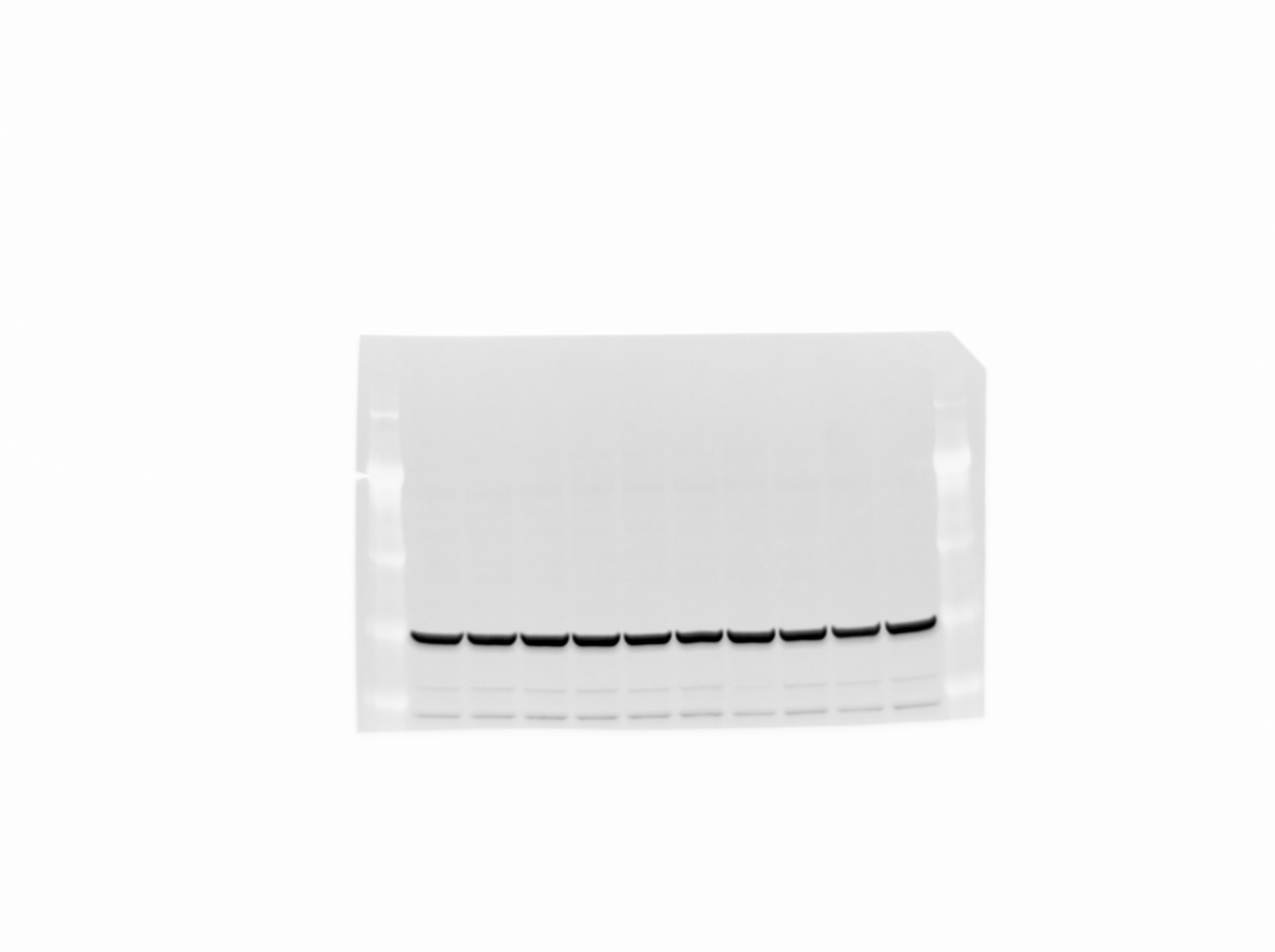

Supplement: Source data 1. [file elife-69207-supp1.zip › Uncropped membrane/Figure 2/Tubulin.tif]

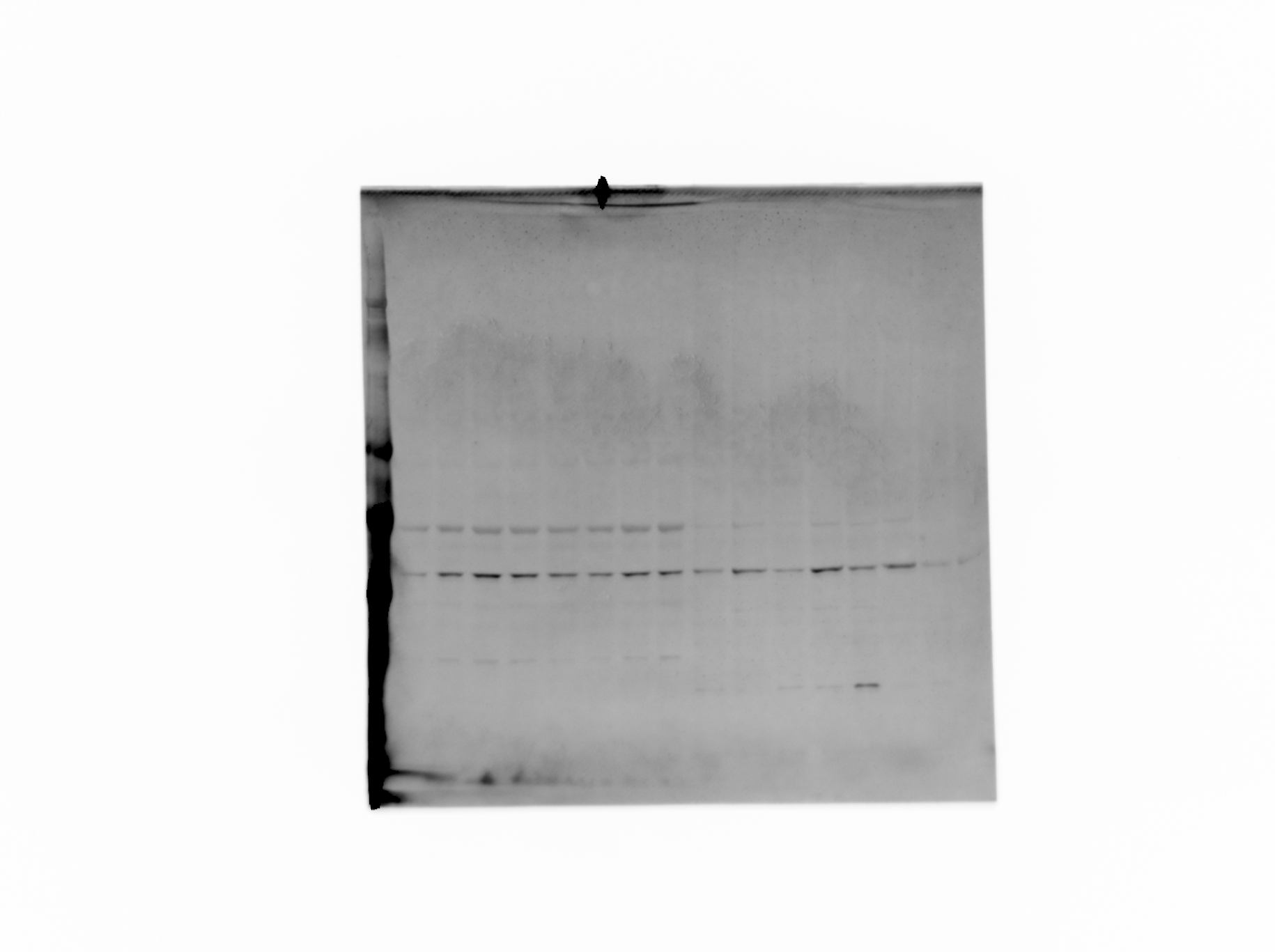

Supplement: Source data 1. [file elife-69207-supp1.zip › Uncropped membrane/Figure 3/Actin.tif]

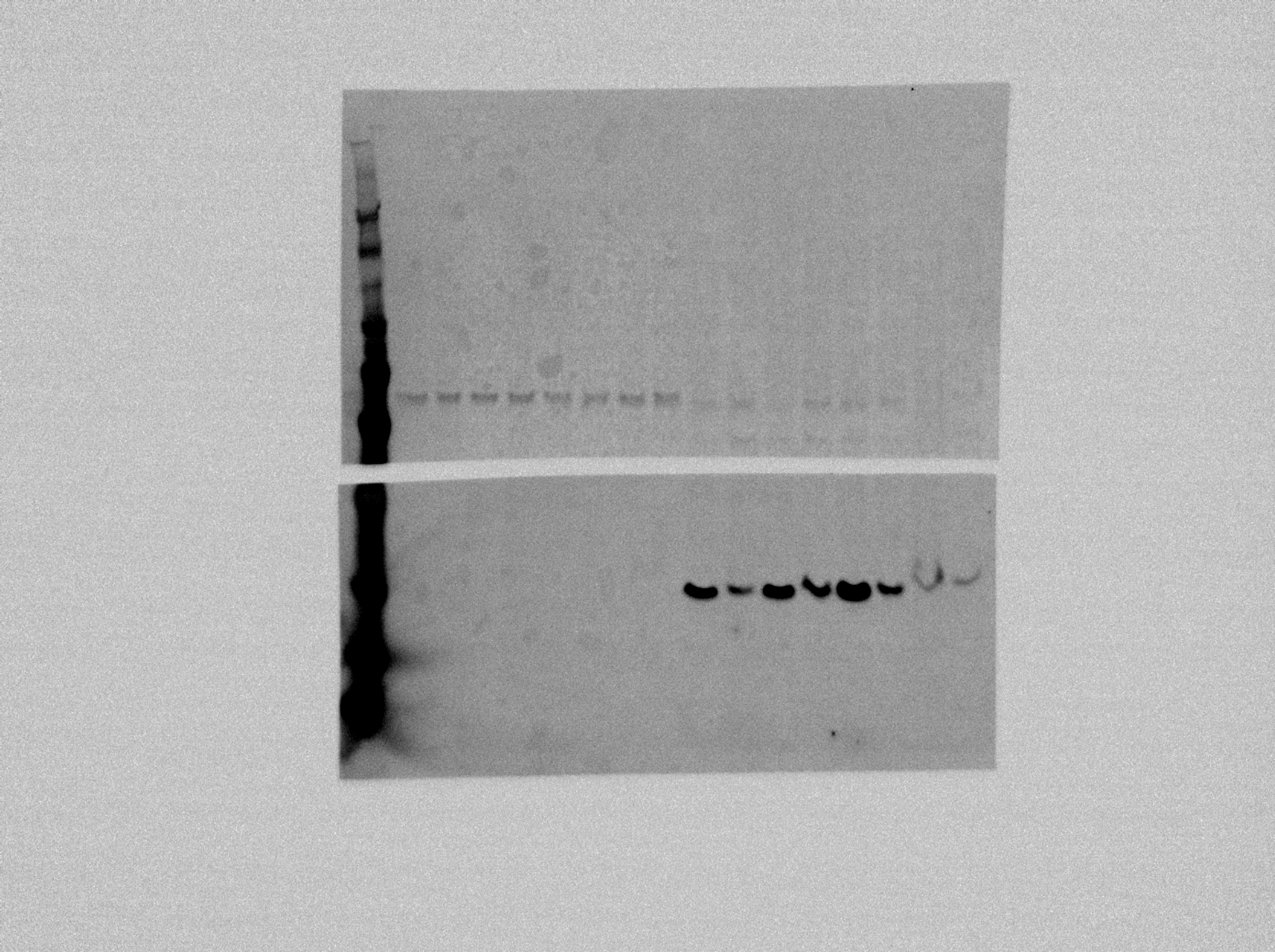

Supplement: Source data 1. [file elife-69207-supp1.zip › Uncropped membrane/Figure 3/COXIV.tif]

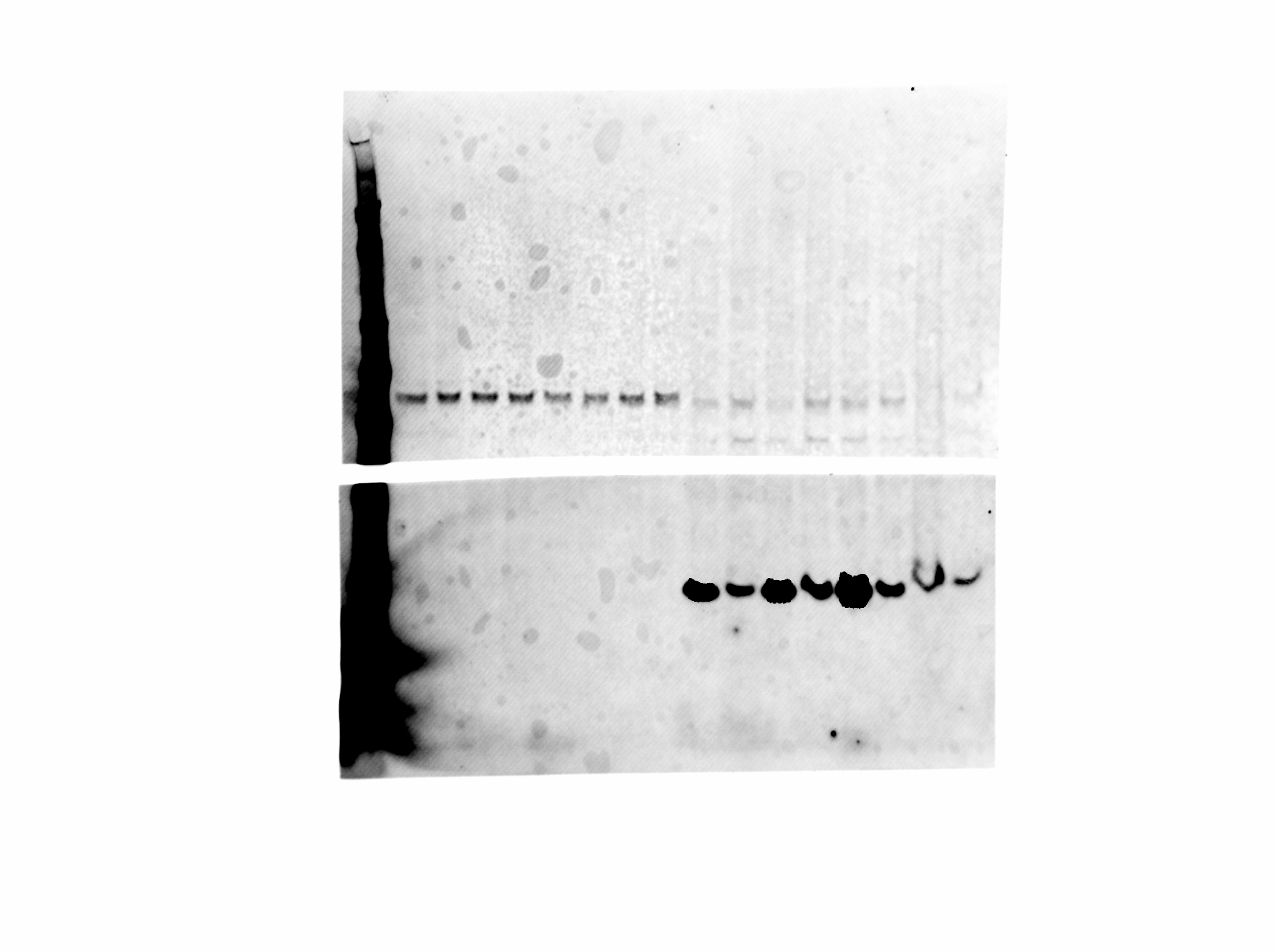

Supplement: Source data 1. [file elife-69207-supp1.zip › Uncropped membrane/Figure 3/MEK1,2.tif]

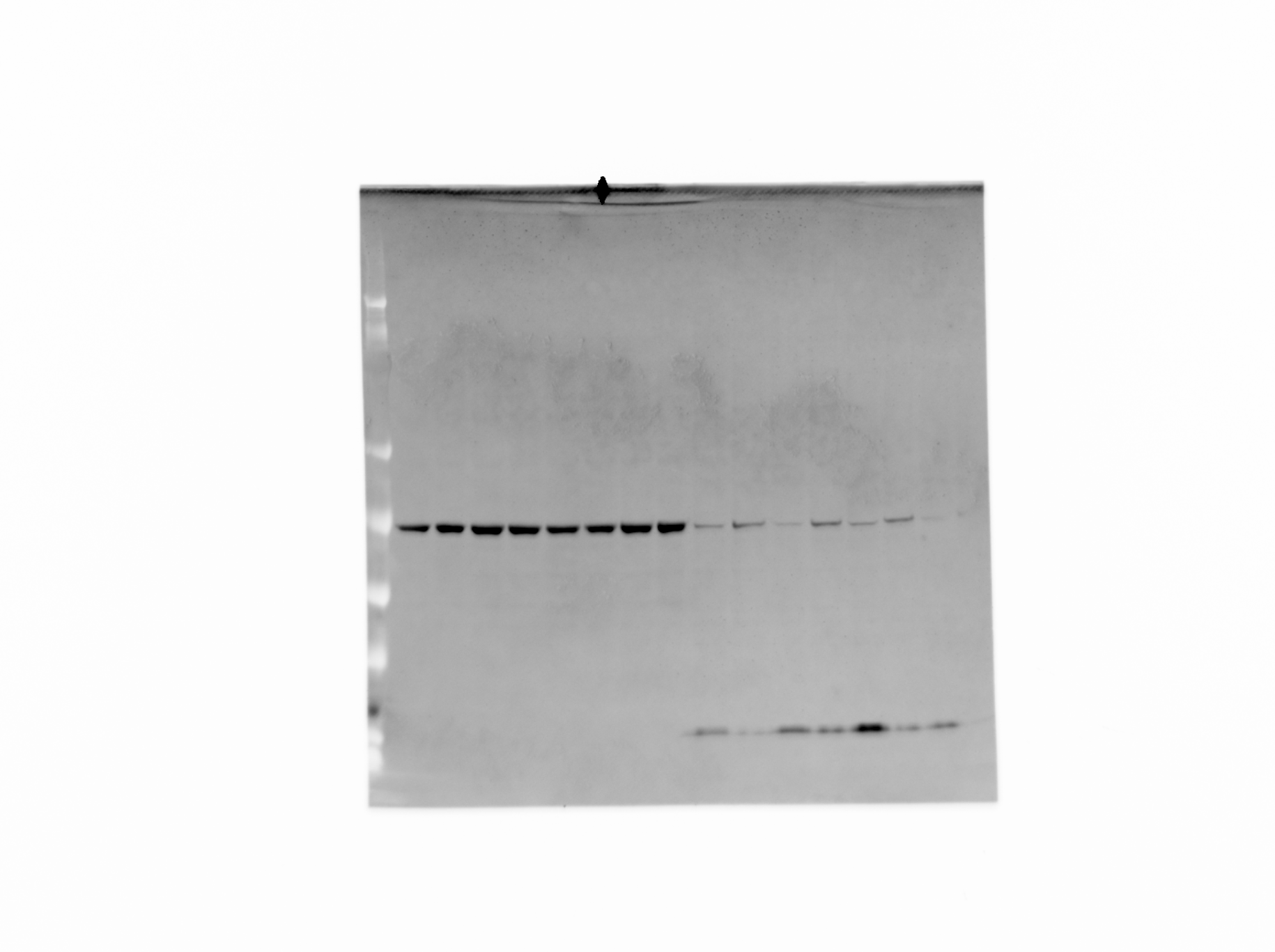

Supplement: Source data 1. [file elife-69207-supp1.zip › Uncropped membrane/Figure 3/tom20.tif]

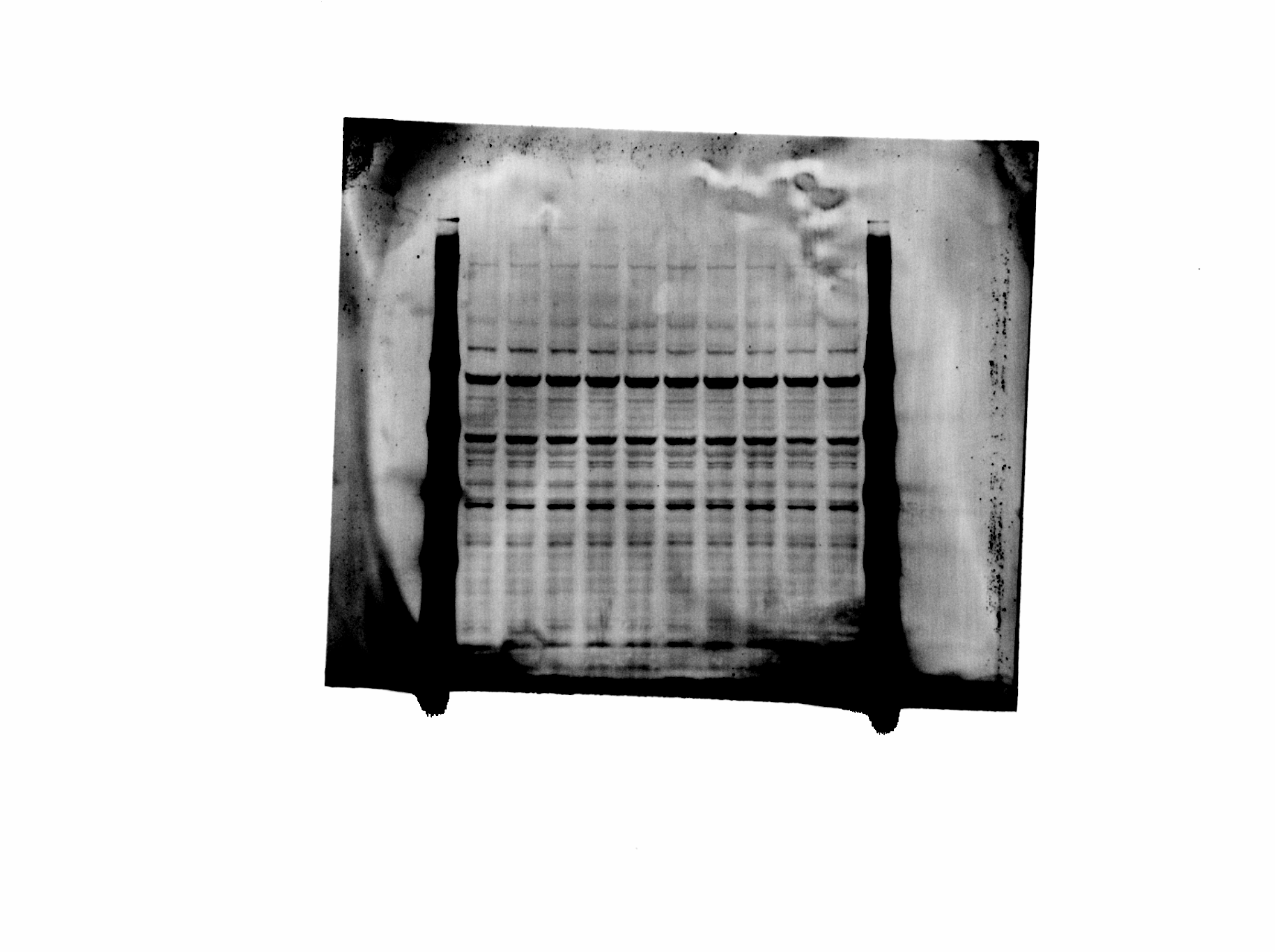

Supplement: Source data 1. [file elife-69207-supp1.zip › Uncropped membrane/Figure 2/p-DRP1(S637).tif]

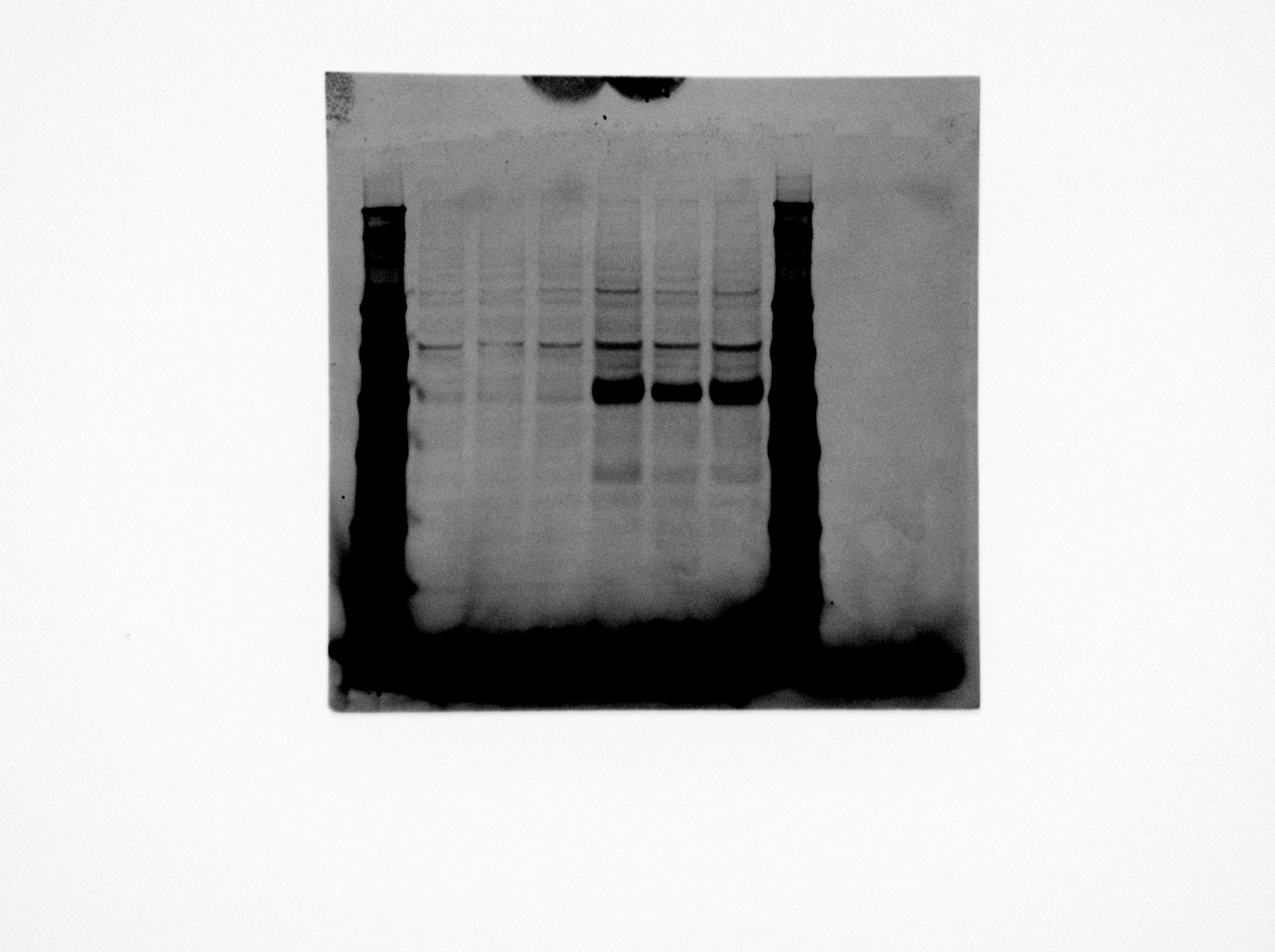

Supplement: Source data 1. [file elife-69207-supp1.zip › Uncropped membrane/Fig1-supplement1/Cx43.tif]

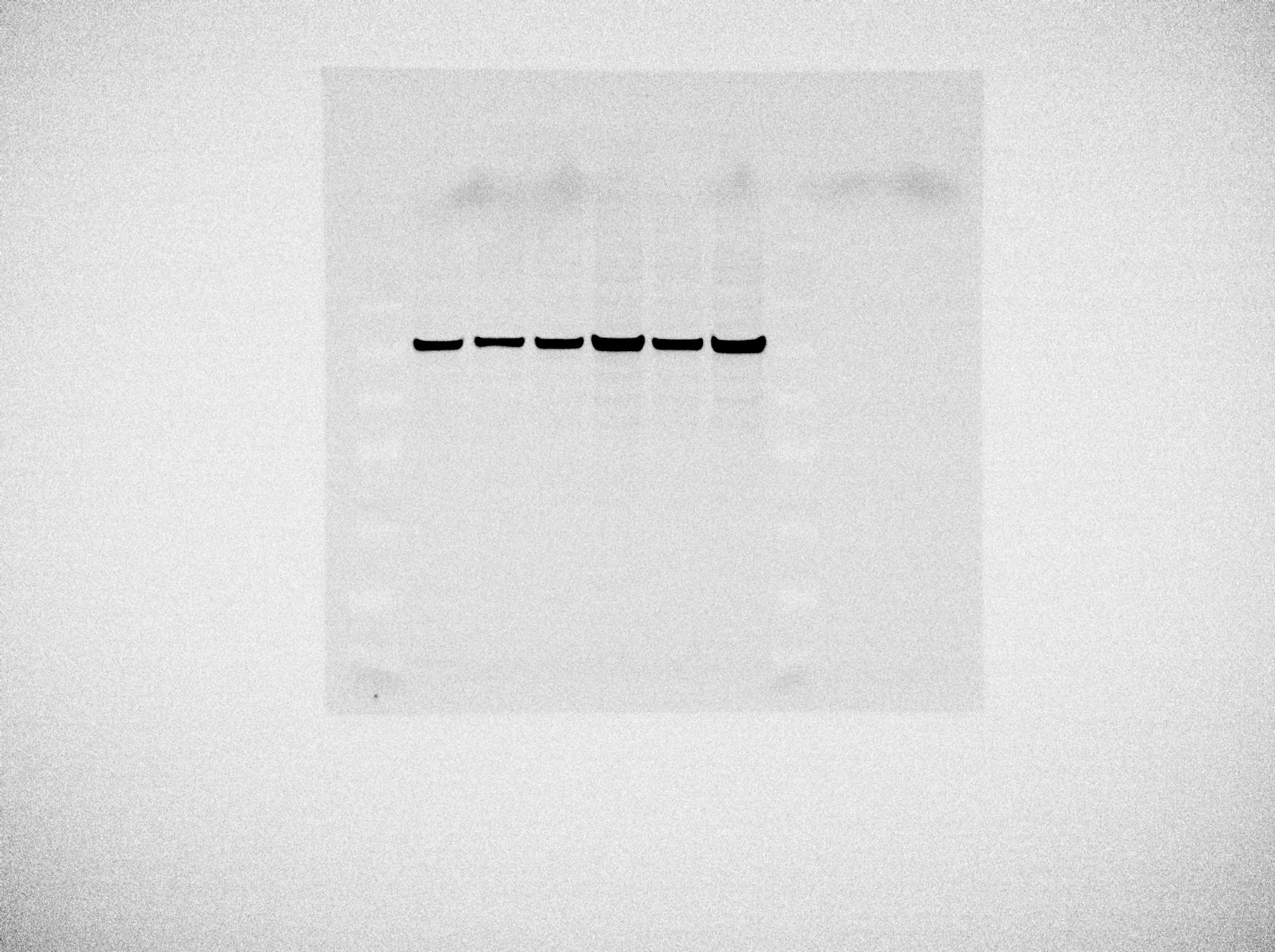

Supplement: Source data 1. [file elife-69207-supp1.zip › Uncropped membrane/Fig1-supplement1/Tubulin.tif]

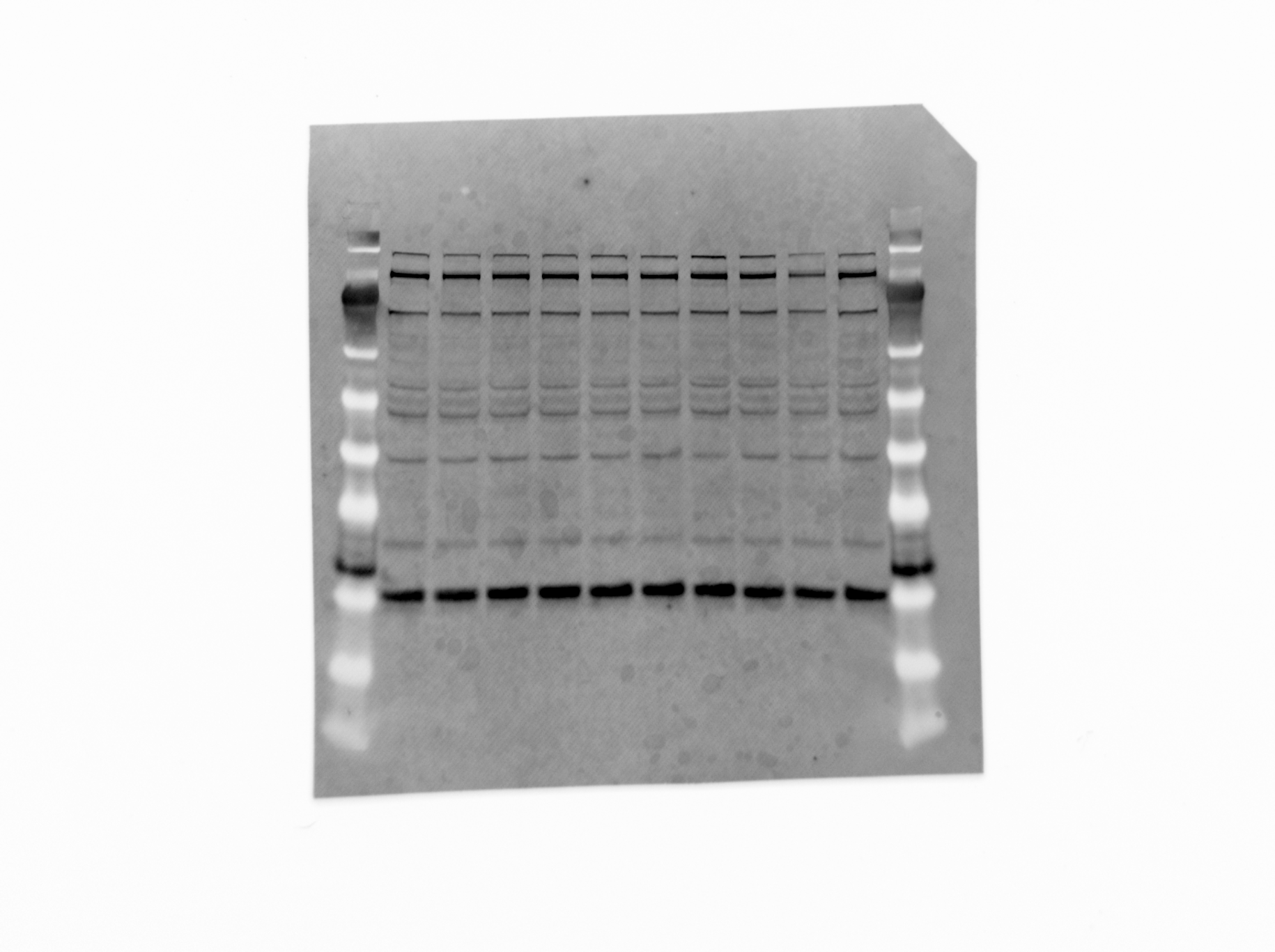

Supplement: Source data 1. [file elife-69207-supp1.zip › Uncropped membrane/Fig1-supplement2/LC3(HEK).tif]

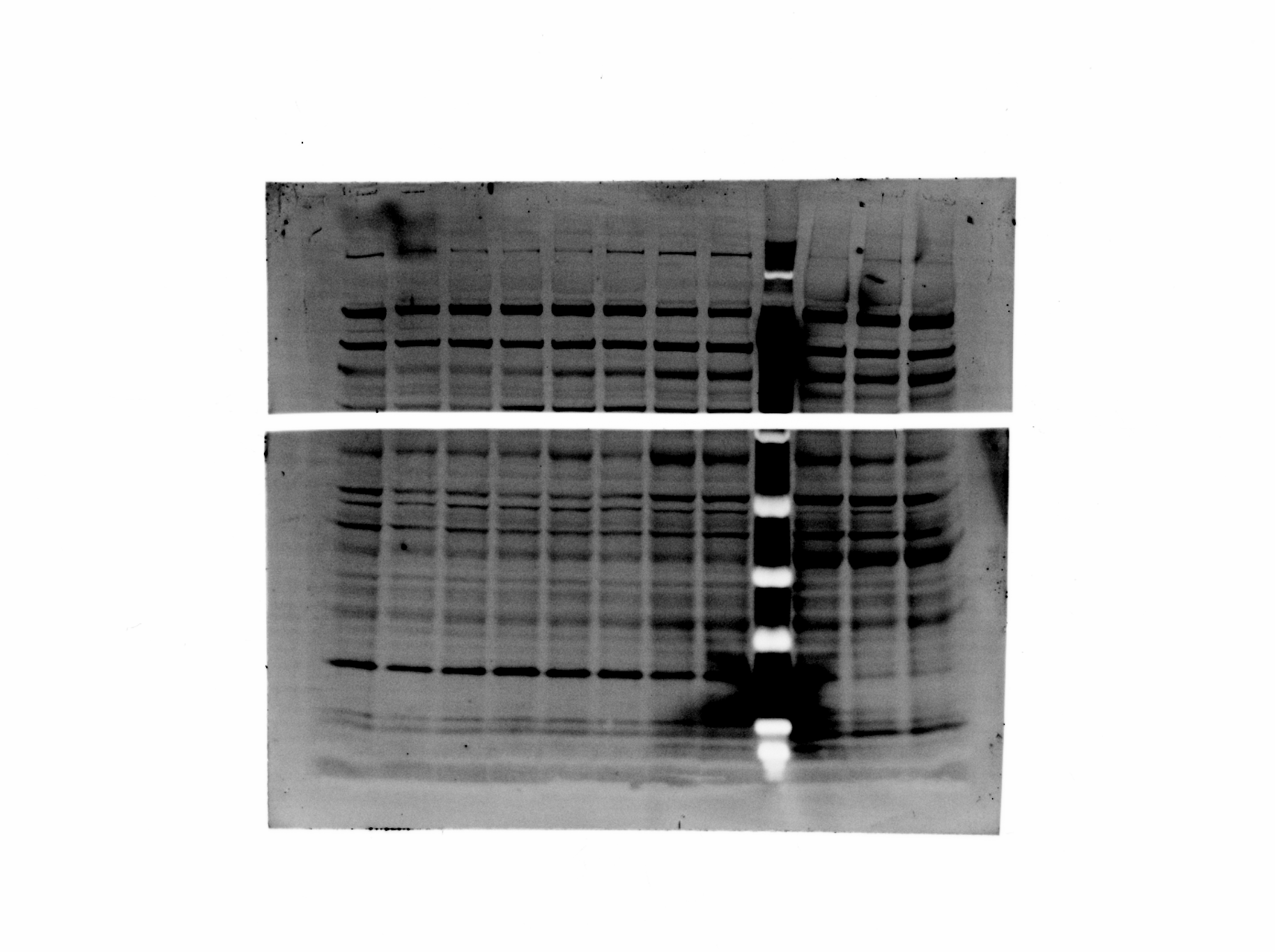

Supplement: Source data 1. [file elife-69207-supp1.zip › Uncropped membrane/Fig1-supplement2/LC3(M213L).tif]

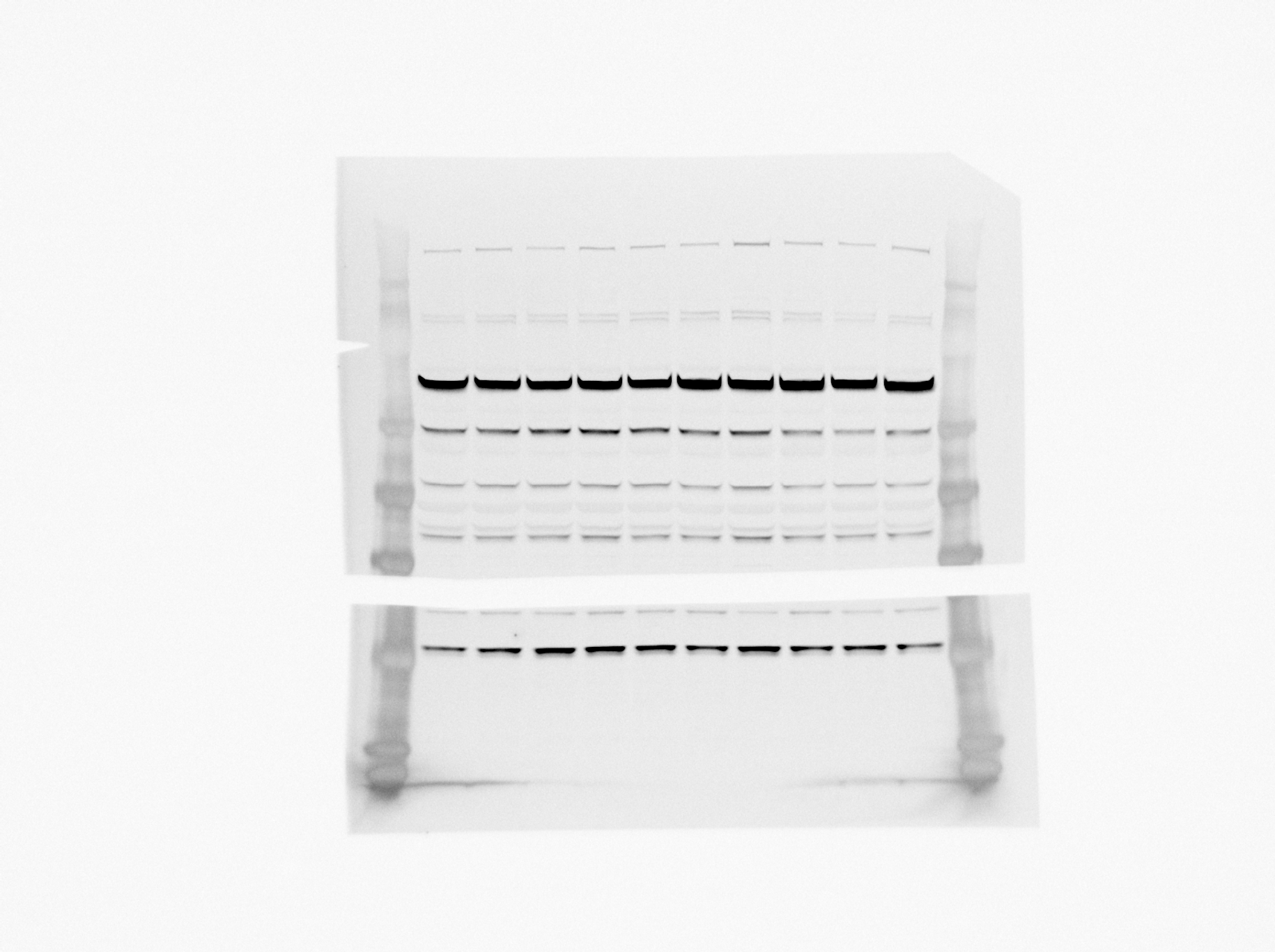

Supplement: Source data 1. [file elife-69207-supp1.zip › Uncropped membrane/Fig1-supplement2/mtTFA(HEK).tif]

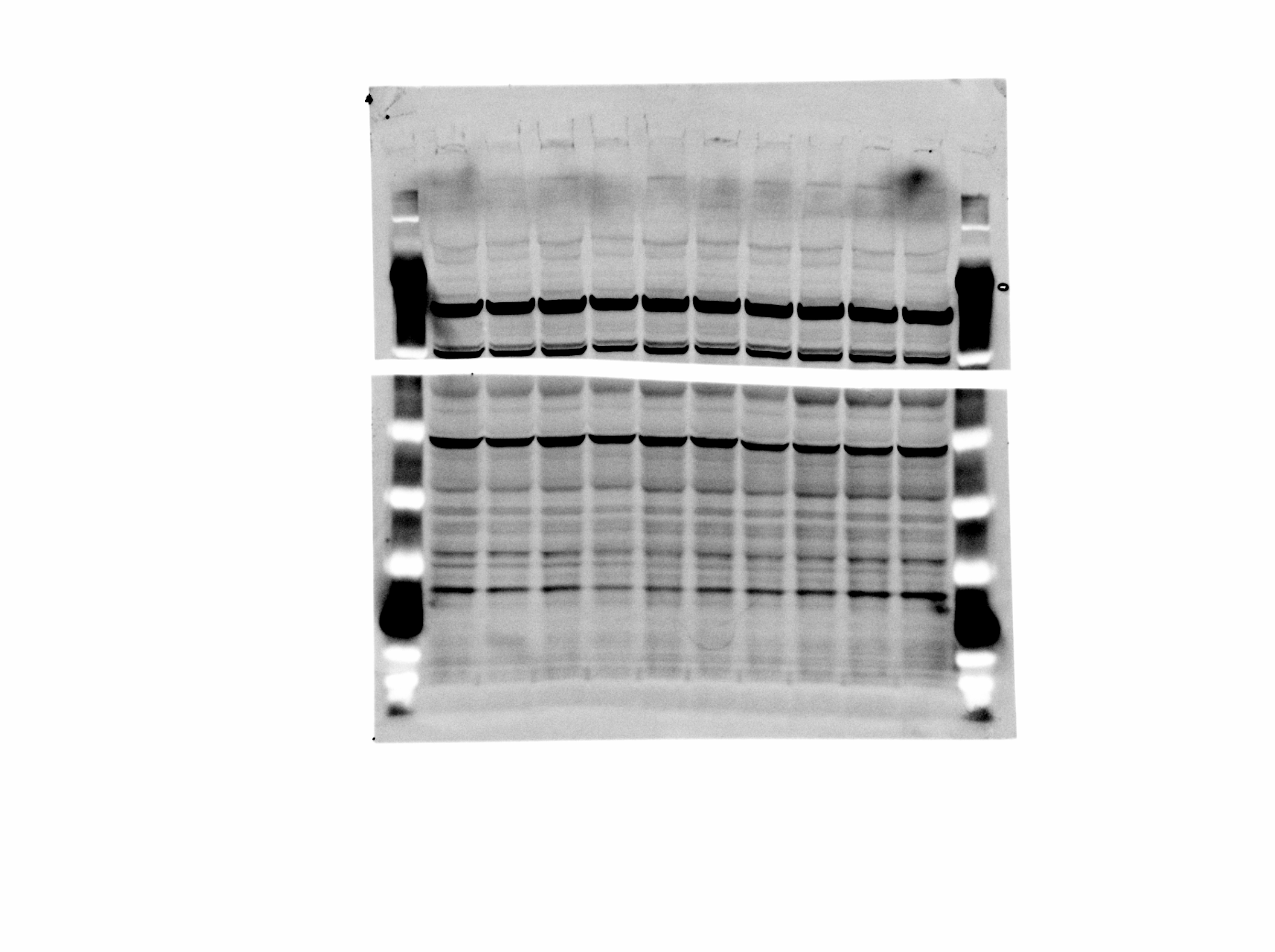

Supplement: Source data 1. [file elife-69207-supp1.zip › Uncropped membrane/Fig1-supplement2/mtTFA(M213L).tif]

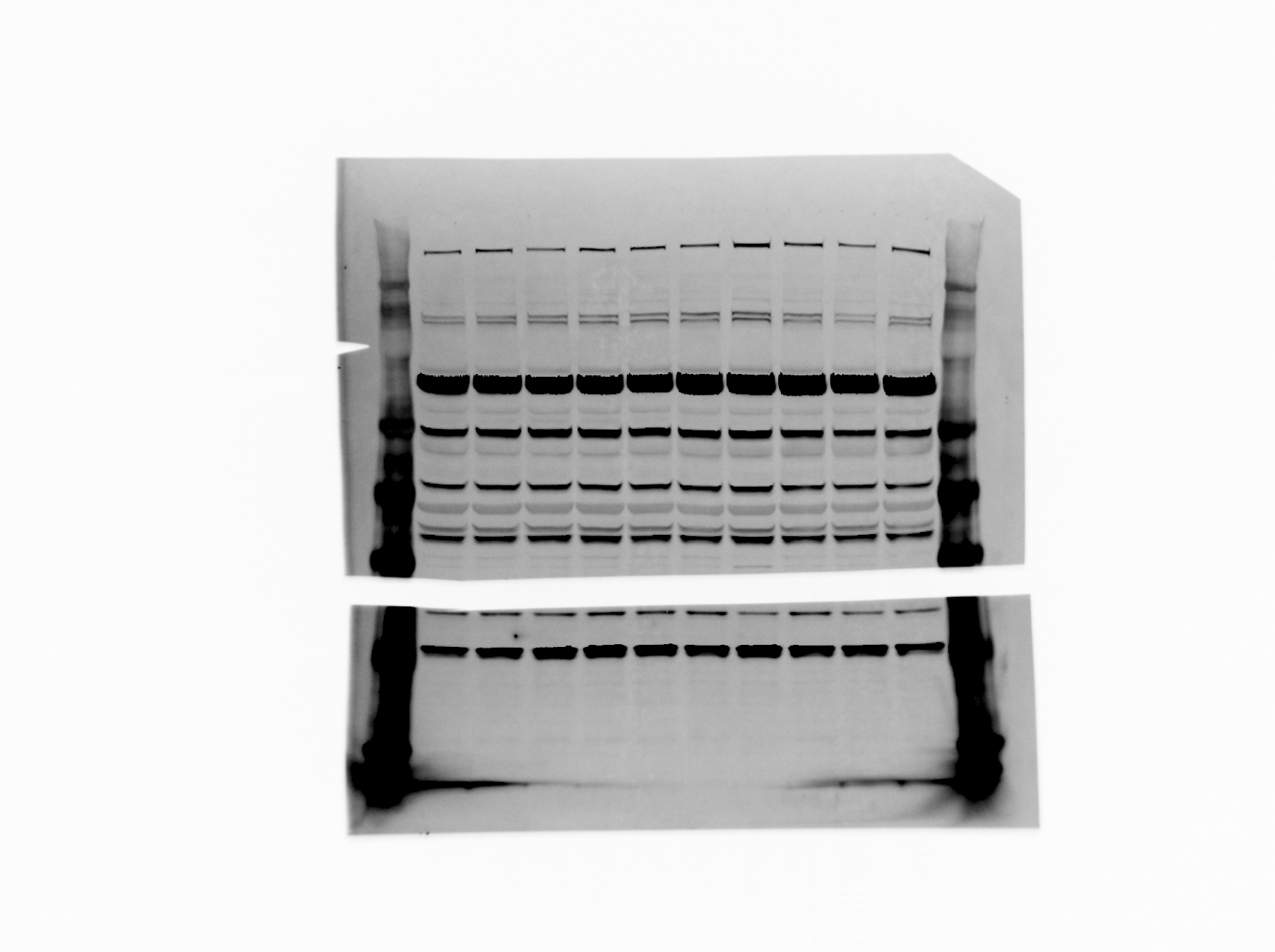

Supplement: Source data 1. [file elife-69207-supp1.zip › Uncropped membrane/Fig1-supplement2/PGC1a(HEK).tif]

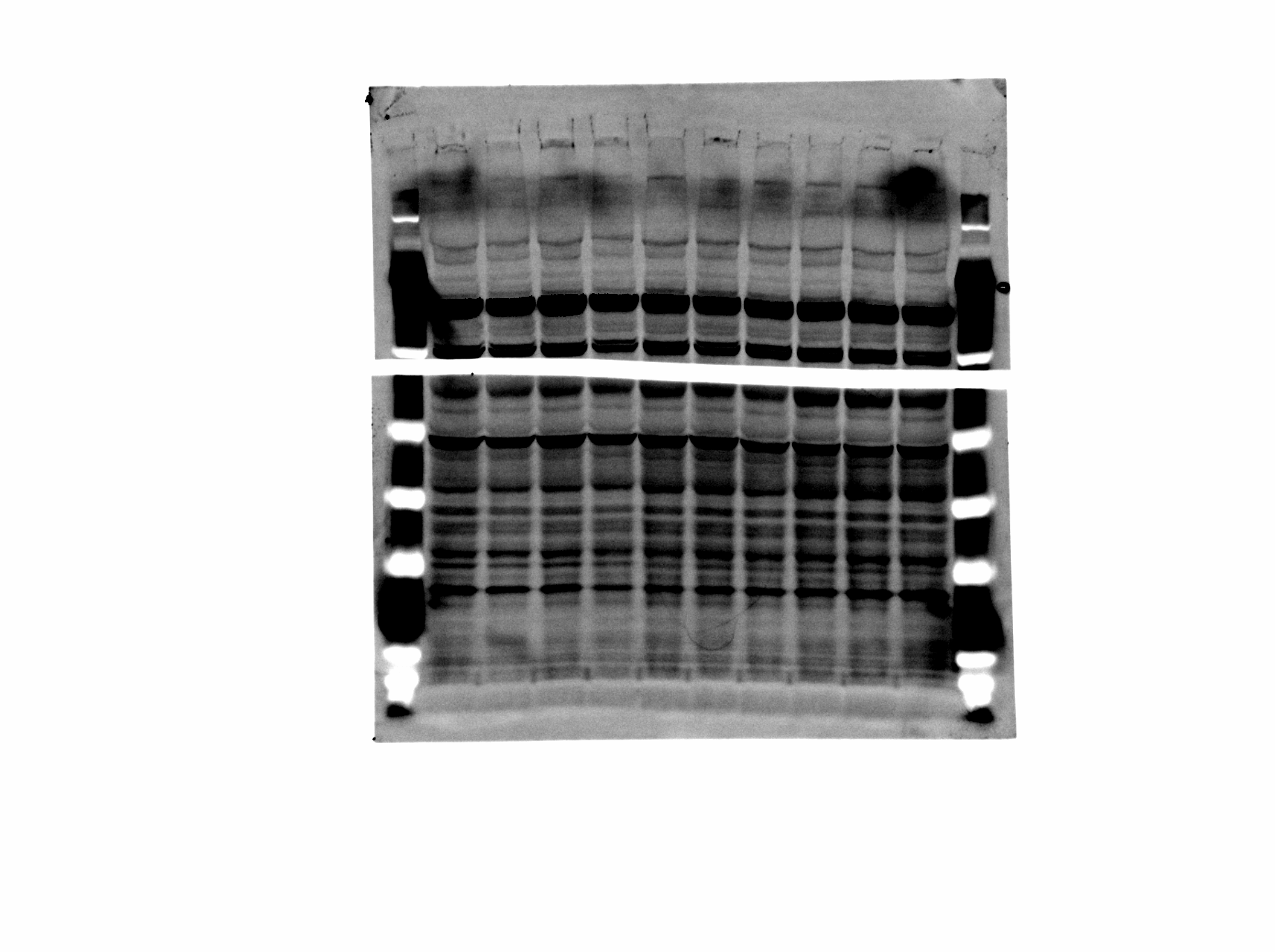

Supplement: Source data 1. [file elife-69207-supp1.zip › Uncropped membrane/Fig1-supplement2/PGC1a(M213L).tif]

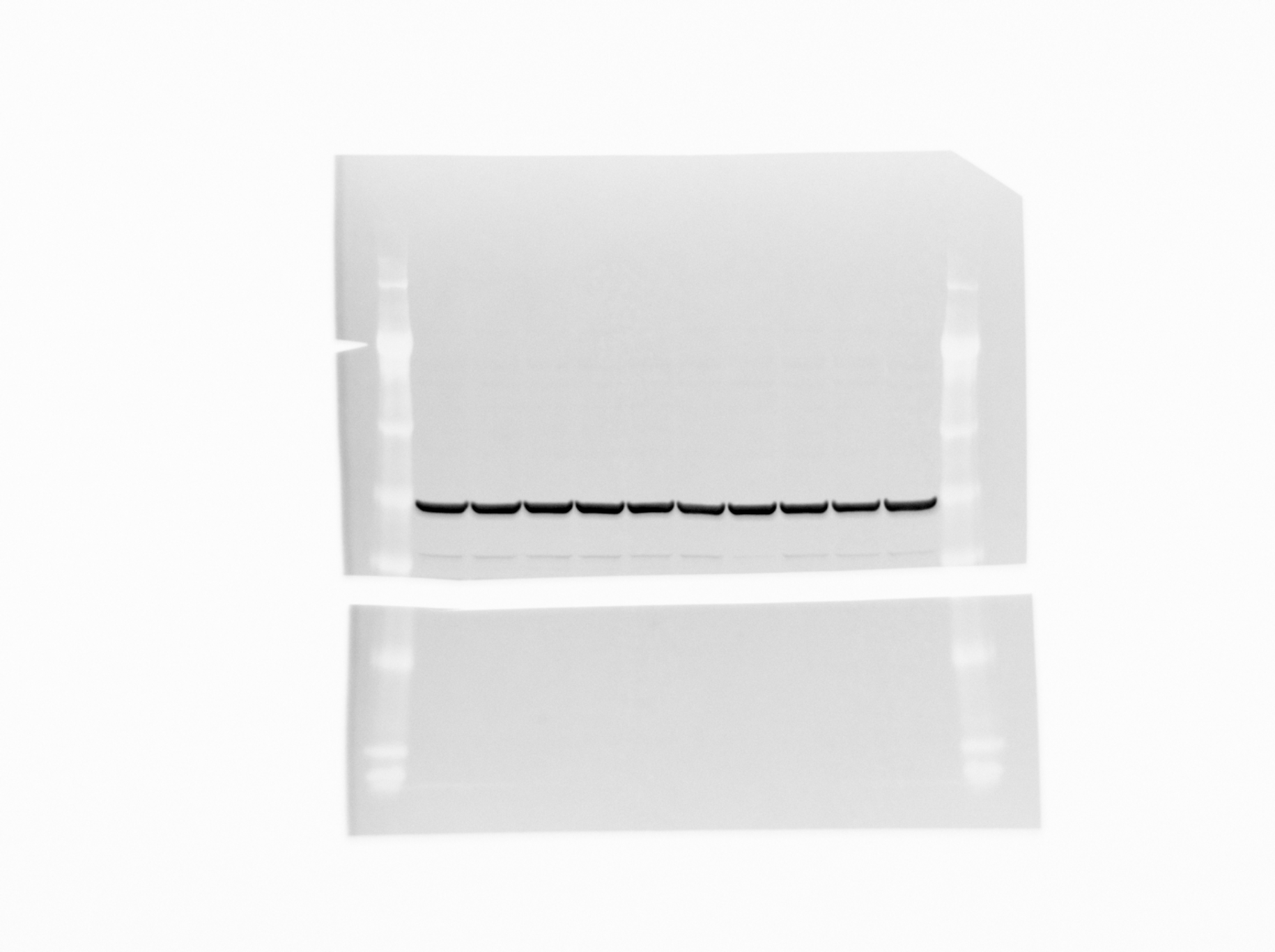

Supplement: Source data 1. [file elife-69207-supp1.zip › Uncropped membrane/Fig1-supplement2/Tubulin(HEK).tif]

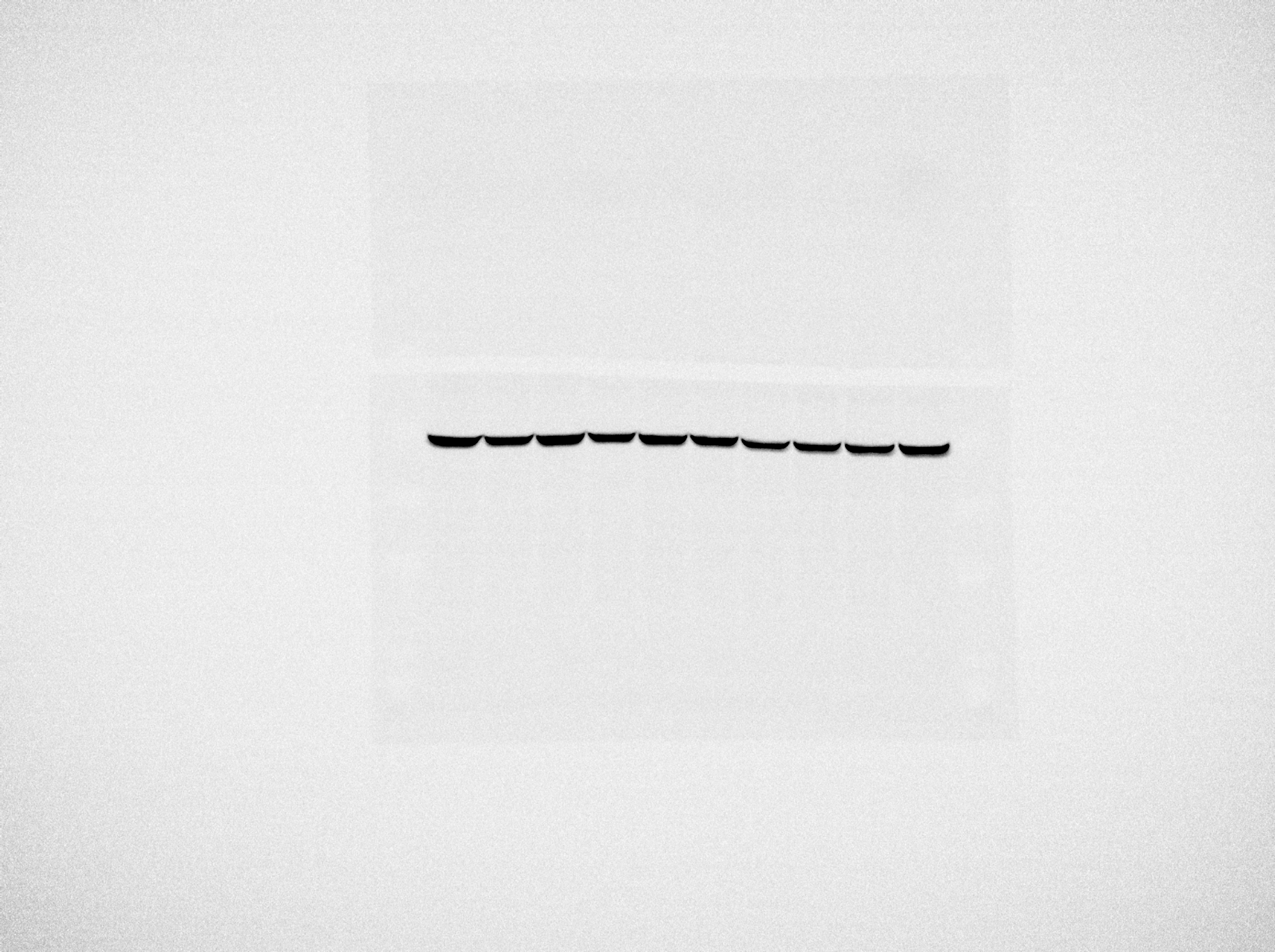

Supplement: Source data 1. [file elife-69207-supp1.zip › Uncropped membrane/Fig1-supplement2/Tubulin(M213L).tif]

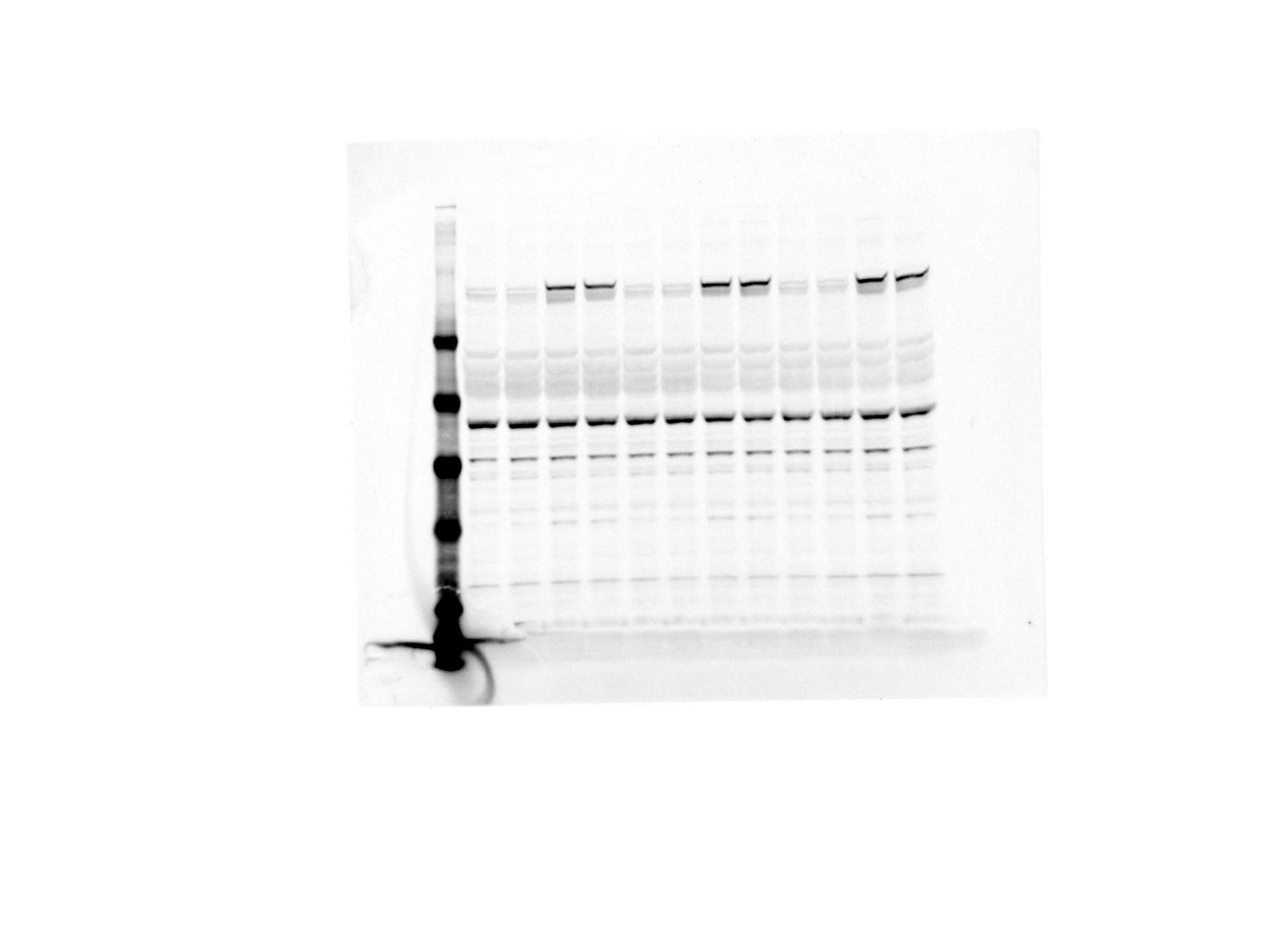

Supplement: Source data 1. [file elife-69207-supp1.zip › Uncropped membrane/Fig2-supplement/siDNM2.tif]

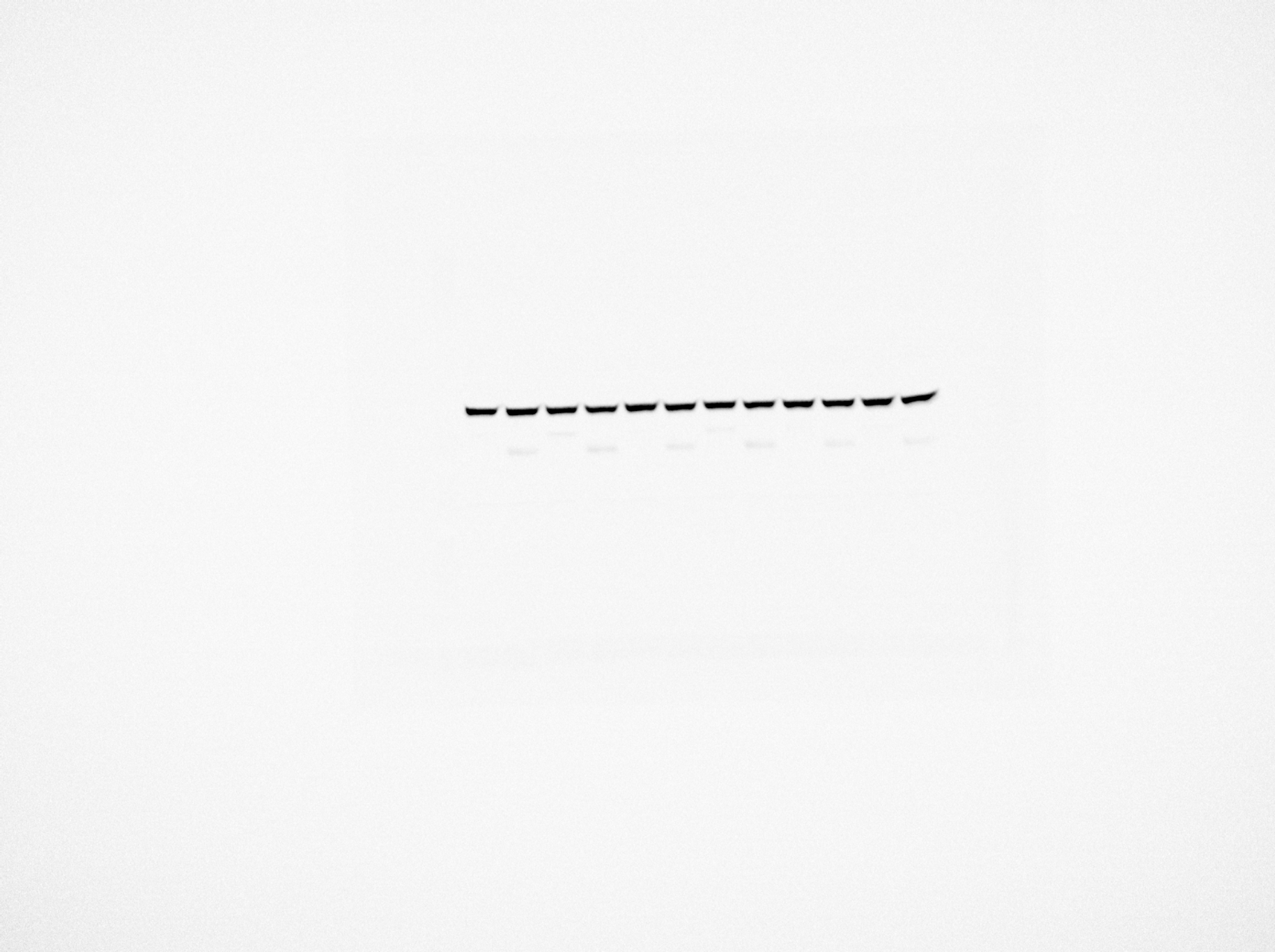

Supplement: Source data 1. [file elife-69207-supp1.zip › Uncropped membrane/Fig2-supplement/tubulin(siDNM2).tif]
